# INHBA(+) cancer-associated fibroblasts generate an immunosuppressive tumor microenvironment in ovarian cancer

**DOI:** 10.1038/s41698-024-00523-y

**Published:** 2024-02-15

**Authors:** Ye Hu, Maria Sol Recouvreux, Marcela Haro, Enes Taylan, Barbie Taylor-Harding, Ann E. Walts, Beth Y. Karlan, Sandra Orsulic

**Affiliations:** 1 Department of Obstetrics and Gynecology, David Geffen School of Medicine, University of California Los Angeles, Los Angeles, CA 90095 USA; 2 Women’s Cancer Program, Samuel Oschin Comprehensive Cancer Institute, Cedars-Sinai Medical Center, Los Angeles, CA 90048 USA; 3 Department of Pathology and Laboratory Medicine, Cedars-Sinai Medical Center, Los Angeles, CA 90048 USA; 4 Jonsson Comprehensive Cancer Center, University of California Los Angeles, Los Angeles, CA 90095 USA; 5 Department of Pathology and Laboratory Medicine, David Geffen School of Medicine, University of California Los Angeles, Los Angeles, CA 90095 USA; 6 United States Department of Veterans Affairs, Greater Los Angeles Healthcare System, Los Angeles, CA 90073 USA; 7 Present Address: Department of Gastroenterology, Xinhua Hospital, Shanghai Jiao Tong University School of Medicine, Shanghai, 200092 China

**Keywords:** Gynaecological cancer, Cell biology

## Abstract

Effective targeting of cancer-associated fibroblasts (CAFs) is hindered by the lack of specific biomarkers and a poor understanding of the mechanisms by which different populations of CAFs contribute to cancer progression. While the role of TGFβ in CAFs is well-studied, less attention has been focused on a structurally and functionally similar protein, Activin A (encoded by INHBA). Here, we identified INHBA(+) CAFs as key players in tumor promotion and immunosuppression. Spatiotemporal analyses of patient-matched primary, metastatic, and recurrent ovarian carcinomas revealed that aggressive metastatic tumors enriched in INHBA(+) CAFs were also enriched in regulatory T cells (Tregs). In ovarian cancer mouse models, intraperitoneal injection of the Activin A neutralizing antibody attenuated tumor progression and infiltration with pro-tumorigenic subsets of myofibroblasts and macrophages. Downregulation of INHBA in human ovarian CAFs inhibited pro-tumorigenic CAF functions. Co-culture of human ovarian CAFs and T cells revealed the dependence of Treg differentiation on direct contact with INHBA(+) CAFs. Mechanistically, INHBA/recombinant Activin A in CAFs induced the autocrine expression of PD-L1 through SMAD2-dependent signaling, which promoted Treg differentiation. Collectively, our study identified an INHBA(+) subset of immunomodulatory pro-tumoral CAFs as a potential therapeutic target in advanced ovarian cancers which typically show a poor response to immunotherapy.

## Introduction

In solid malignancies, the predominant cell types contributing to the tumor mass are cancer cells, immune cells, and CAFs with their associated extracellular matrix (ECM). Typically, a high content of immune cells is associated with a good prognosis while a high content of CAFs is associated with a poor prognosis. However, tumors enriched for CAFs are often also enriched for immune cells that reside within the ECM. In such cases, clinical outcomes are influenced by the proportions of immune cells vs. CAFs and the spatial distribution and functional properties of these cell types^1–3^. CAFs can influence the behavior of cancer cells and immune cells through biochemical and biomechanical signals^1–3^. CAF activation is associated with secretion of ECM, chemokines, cytokines, and growth factors^1–3^. CAF-secreted ECM contributes to therapeutic resistance by blocking access to chemotherapies and immunotherapies and hindering the trafficking of functional immune cells to the tumor bed^4–6^. ECM is also a rich source of biologically active molecules that attract or repel certain immune cell types and modify immune cell function^7^.

Among the biologically active molecules, TGFβ stands out as a key inducer of genes and proteins involved in ECM remodeling^4–6^. Beyond its role in ECM dynamics, TGFβ exerts a potent influence on the function of immune cells^4–6^. Studies in several cancer types have shown that the expression of TGFβ-associated ECM genes in CAFs was the strongest predictor of failure in immunotherapeutic interventions^8^. Consequently, many preclinical and clinical studies have focused on blocking TGFβ signaling in cancer^9^. In mouse models of breast and colon cancers, various methods of TGFβ inhibition increased the efficacy of PD-1/PD-L1 immunotherapies by reducing TGFβ signaling in stromal cells thereby facilitating CD8(+) T-cell penetration into the tumor and provoking robust anti-tumor immunity, ultimately leading to tumor regression^4–6^. However, targeting TGFβ has been associated with cardiovascular toxicity and the development of benign tumors, possibly because TGFβ is a pleiotropic cytokine expressed by multiple stromal cell types, some of which are required for normal tissue homeostasis^9^. Variable success in enhancing immunotherapeutic efficacy by targeting TGFβ underscores the need to better understand the complexity of TGFβ signaling in order to develop more precise and less toxic therapies for targeting the tumor stroma.

Activin A, a member of the TGFβ superfamily, has received comparatively less attention despite its structural resemblance to TGFβ and the shared canonical SMAD2/3 pathway. This structural similarity implies overlapping functions between Activin A and TGFβ. In instances where TGFβ signaling is compromised, Activin A has the capacity to step in and compensate for the deficiency in SMAD2/3 phosphorylation. This compensatory mechanism enables Activin A to play a crucial role in sustaining cellular homeostasis and fundamental cellular functions^10^. Importantly, like TGFβ, Activin A was shown to inhibit the function of dendritic cells (DC)^11^ and promote T regulatory cell (Treg)-mediated immunosuppression in allergic disorders^12–14^ and cancer^15^. A study of the effect of CTLA-4 immunotherapy in a mouse model of breast cancer showed that the TGFβ blockade induced Foxp3 expression and generation of Tregs through a compensatory increase in Activin A signaling^15^. A concomitant blockade of both TGFβ and Activin A was necessary to achieve a durable immune response with CTLA-4 immunotherapy in this model^15^.

## Results

### INHBA is expressed in a subset of α-SMA(+) CAFs that are enriched during ovarian cancer progression

INHBA mRNA is upregulated in multiple cancer types in comparison to normal tissues (Supplementary Fig. 1). INHBA gene encodes the Inhibin βA homodimer of Activin A. Genes encoding related protein subunits, such as INHBB, INHBC, INHBE, and INHA, are not overexpressed in cancers (Supplementary Fig. 1), suggesting that upregulation of INHBA in cancer leads to increased production of Activin A (a homodimer of two Inhibin βA subunits) but not of Inhibin A (a heterodimer of Inhibin βA and Inhibin α subunits) or of other heterodimeric proteins containing the Inhibin βA subunit (Supplementary Fig. 1). Consistent with these data, only INHBA expression is associated with poor overall and disease-free survival in ovarian cancer patients (Supplementary Fig. 2a) while genes encoding other subunits are not associated with survival.

Although INHBA is seldomly expressed in epithelial cancer cell lines, the majority of cancer-related research on INHBA has utilized rare epithelial cancer cell lines that express INHBA or cancer cell lines in which INHBA was ectopically overexpressed^15–25^. Additionally, uncertainty regarding the origin of INHBA overexpression in solid tumors has arisen due to the use of poorly-validated INHBA antibodies in the analysis of cancer tissues. These antibodies have led to ambiguous results, suggesting the presence of Inhibin βA protein in both epithelial cancer cells and fibroblasts^16,24,26–28^. Notably, studies in colorectal cancer revealed a tenfold higher expression of Activin A in cancer-associated fibroblasts (CAFs) compared to epithelial cancer cells^29,30^. In pancreatic cancer, only stromal Activin A expression correlated with poor survival while epithelial expression had no correlation with disease outcome^27^. Such observations raise concerns about the specificity of the Activin A antibodies. To accurately identify cell types that express INHBA in human and mouse ovarian tumors, we used in situ hybridization (ISH) with human and mouse INHBA probes, respectively. We found that INHBA mRNA was expressed exclusively in CAFs in human HGSOC (Supplementary Fig. 3a) and the BR-luc syngeneic mouse ovarian cancer model (Supplementary Fig. 3b). Consistent with the ISH data, INHBA levels in the microarray dataset of laser-capture-microdissected epithelial cells and fibroblasts from normal ovaries and HGSOC^31^ confirmed high levels of INHBA in CAFs with only negligible levels in epithelial cancer cells, normal ovarian surface epithelial cells, and normal ovarian fibroblasts (Supplementary Fig. 3c).

Studies in different cancer types have identified several transcriptionally distinct CAF subtypes^3^. Using a pan-cancer single-cell RNA sequencing profile of the tumor microenvironment^32^, we show that INHBA is expressed in a small subset of ACTA2 (gene encoding for α-SMA)-expressing fibroblasts in HGSOC (Supplementary Fig. 4). In contrast to INHBA, ACTA2 was not associated with survival in ovarian cancer (Supplementary Fig. 2b) and exhibited broad expression in most CAFs (Supplementary Fig. 4). In fibroblasts derived from ovarian, lung, and colorectal cancers and their respective normal tissues, INHBA was specifically expressed in fibroblasts derived from cancer tissues, while ACTA2 was expressed in fibroblasts derived from both cancer and normal tissues (Supplementary Fig. 4) and was largely absent from fibroblasts derived from ascites from ovarian cancer patients (Supplementary Fig. 5). Together, these data show that INHBA is expressed in a subset of ACTA2 (+) CAFs and is only rarely expressed in normal tissue fibroblasts and normal and malignant epithelial cells.

**Fig. 1:**
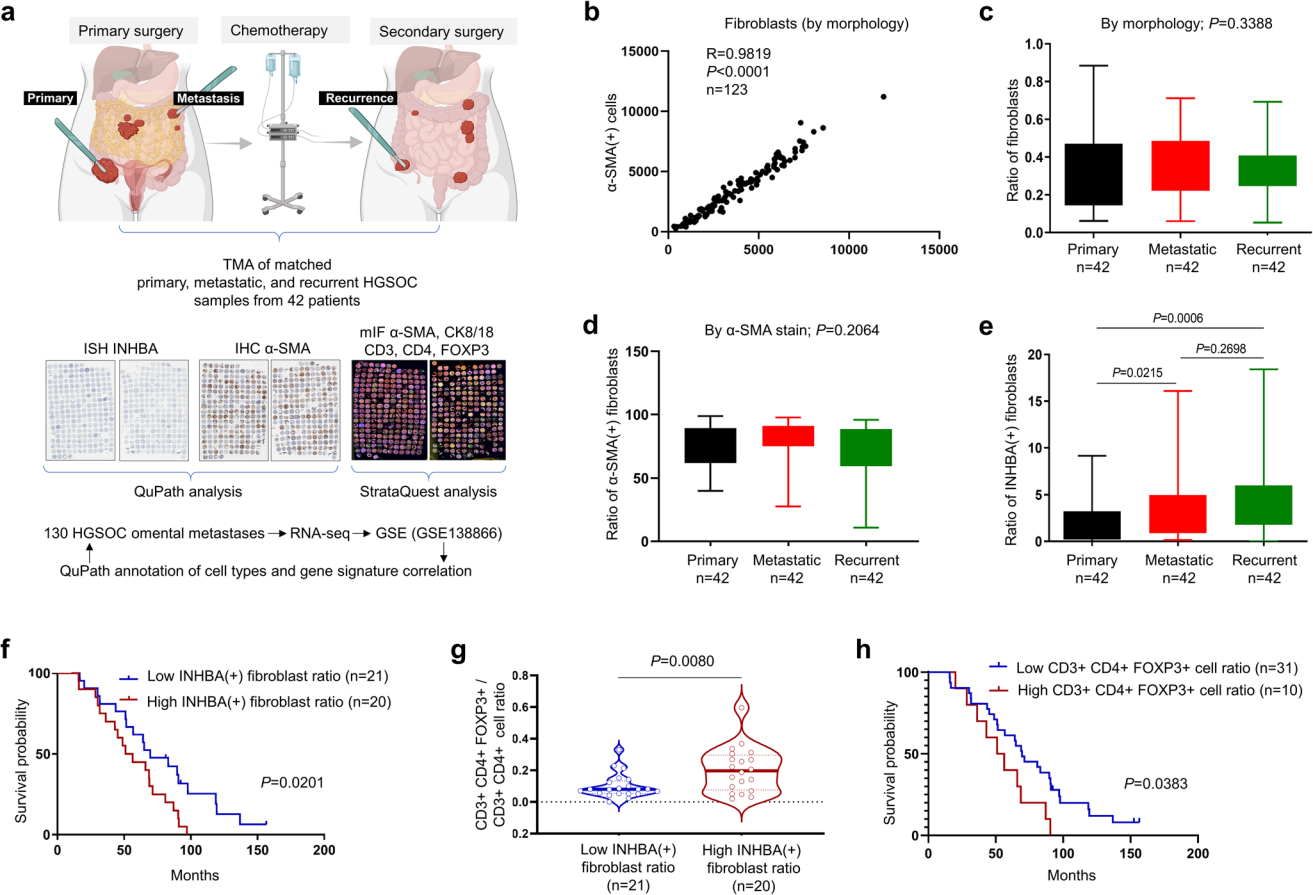
INHBA(+) fibroblasts are enriched in cancer metastases and are associated with Tregs. **a** A schematic of the process of image analysis in the TMA of patient-matched primary, synchronous metastatic, and metachronous recurrent HGSOC samples from 42 patients. The image was generated using BioRender.com. Each primary, metastatic, and recurrent HGSOC sample is represented by 3 cores that were punched at different locations in the corresponding original FFPE tumor block. The 378 cores were distributed on two slides. INHBA was detected by ISH while α-SMA and immune markers were detected by IHC. **b** Correlation between the number of fibroblasts (defined by morphology-based classification) and α-SMA(+) cells in the TMA (*P* < 0.0001, Pearson correlation, *n* = 123; 3 cores were excluded because of insufficient material for analysis). **c** Statisti**c**al analysis of the ratio of fibroblasts (defined by morphology-based classification) in primary, metastatic, and recurrent tumors (ANOVA test, *P* = 0.3388). **d** Statistical analysis of the ratio of α-SMA(+) fibroblasts in primary, metastatic, and recurrent tumors (ANOVA test, *P* = 0.1064). **e** Statistical analysis of the proportion of INHBA(+) fibroblasts (paired *t* test). In all box-and-whisker plots, boxes indicate the 25th to 75th percentiles, and whiskers indicate the minimum and maximum values. **f** Kaplan-Meier survival plot comparison between patients with a low vs high ratio of INHBA(+) fibroblasts (log-rank test). **g** Treg cell ratio (CD3( + ) CD4( + ) FOXP3(+) cells / CD3(+) CD4(+) cells) in the fibroblast area of HGSOC omental metastasis samples with a low vs high ratio of INHBA(+) fibroblasts (Mann-Whitney U test). **h** Kaplan-Meier survival plot comparison between patients with a low vs. high Treg ratio in samples of HGSOC metastases (log-rank test). In (B-E), data from 1-3 available cores for each sample were averaged. TMA, tissue microarray; ISH, in situ hybridization; IHC, immunohistochemistry; mIF, multiplex immunofluorescence.

To comprehensively quantify INHBA(+) CAFs during HGSOC progression, we performed INHBA ISH, α-SMA immunohistochemistry (IHC), and multiplex immunofluorescence (mIF; α-SMA, cytokeratin 8/18, CD3, CD4, CD8, and FOXP3) staining in a tissue microarray (TMA) consisting of matched primary, synchronous metastatic, and metachronous recurrent cancer samples from 42 HGSOC patients (Fig. 1a, Supplementary Fig. 6 and Supplementary Fig. 7). Quantitative Pathology & Bioimage Analysis (QuPath) machine learning software was used to identify fibroblasts and epithelial cancer cells by morphologic features (Fig. 1a). The correlation coefficient between α-SMA(+) cells and cells classified by morphology as fibroblasts was 0.98 (Fig. 1b) confirming that the QuPath learning algorithm can accurately identify fibroblasts by morphology in H&E sections. There were no significant differences between primary, metastatic, and recurrent samples in the ratio of fibroblasts (number of fibroblasts / number of fibroblasts + cancer cells + immunocytes) (Fig. 1c) and the ratio of α-SMA(+) fibroblasts (number of α-SMA(+) cells / number of fibroblasts) (Fig. 1d). However, the ratio of INHBA(+) fibroblasts (number of INHBA(+) cells / number of fibroblasts) was significantly higher in metastatic and recurrent samples compared to primary tumor samples (Fig. 1e).

To better understand the relationship between INHBA(+) fibroblasts and other cell types in HGSOC metastases, we chose to focus on omental metastases since the omentum is the most common site of HGSOC metastases. We generated RNA-seq transcriptome data (GSE138866) from formalin-fixed paraffin-embedded (FFPE) sections of omental metastases from 130 HGSOC patients^33^ and used QuPath analysis of H&E slides from the same samples to classify different cell types as immunocytes, fibroblasts, epithelial cancer cells and others. We observed no correlation between the ratio of immunocytes and fibroblasts and a slightly negative correlation of immunocytes and epithelial cancer cells (Supplementary Fig. 8a). As an independent confirmation that INHBA is expressed in fibroblasts in human HGSOC, the expression of INHBA was positively correlated with the ratio of fibroblasts, negatively correlated with the ratio of epithelial cancer cells, and not correlated with the ratio of immunocytes (Supplementary Fig. 8b). We then tested if there was a correlation between INHBA expression and specific pro- and anti-tumorigenic subtypes of immunocytes. By applying immune cell metagenes derived from colorectal cancers^34^ and pan-cancer immune cell metagenes^35^ to the transcriptome data of 130 omental HGSOC metastases, we found that INHBA expression positively correlated with metagenes of Tregs but not with metagenes of activated CD4 T cells or activated CD8 T cells (Supplementary Fig. 8c).

Given the enrichment of INHBA(+) fibroblasts in metastatic and recurrent HGSOC in comparison to patient matched primary HGSOC (Fig. 1) and the positive correlation of INHBA expression with metagenes of Tregs in omental metastases (Supplementary Fig. 8c), we tested in a TMA of HGSOC metastases whether stroma with a high content of INHBA(+) fibroblasts also had a high content of Tregs. We used mIF α-SMA staining to generate a fibroblast-positive mask and detect CD3(+), CD4(+), and FOXP3(+) immune cells within the fibroblast-positive mask. Data from FFPE cores of 41 HGSOC metastases were analyzed using the StrataQuest software. Since these samples were not labeled with INHBA, we used INHBA ISH data from the same cores (Fig. 1a) and divided the samples by the average ratio of INHBA(+) fibroblasts into low INHBA(+) fibroblast ratio (*n* = 21) and high INHBA(+) fibroblast ratio (*n* = 20). This division was clinically relevant as patients with a high ratio of INHBA(+) fibroblasts had shorter overall survival (Fig. 1f). The analysis showed that the ratio of Tregs [(CD3(+) CD4(+) FOXP3(+) cells/CD3(+) CD4(+) cells] in the fibroblast-positive mask was significantly higher in samples with a high ratio of INHBA(+) fibroblasts (Fig. 1g). In addition, the high ratio of Tregs was associated with shorter OS of patients in this cohort (Fig. 1h). Together, these data suggest a possible immunoregulatory function of INHBA(+) fibroblasts in human ovarian cancer.

### Interference with INHBA signaling attenuates syngeneic ovarian cancer growth and recruitment of pro-tumorigenic fibroblasts and macrophages

**Fig. 2:**
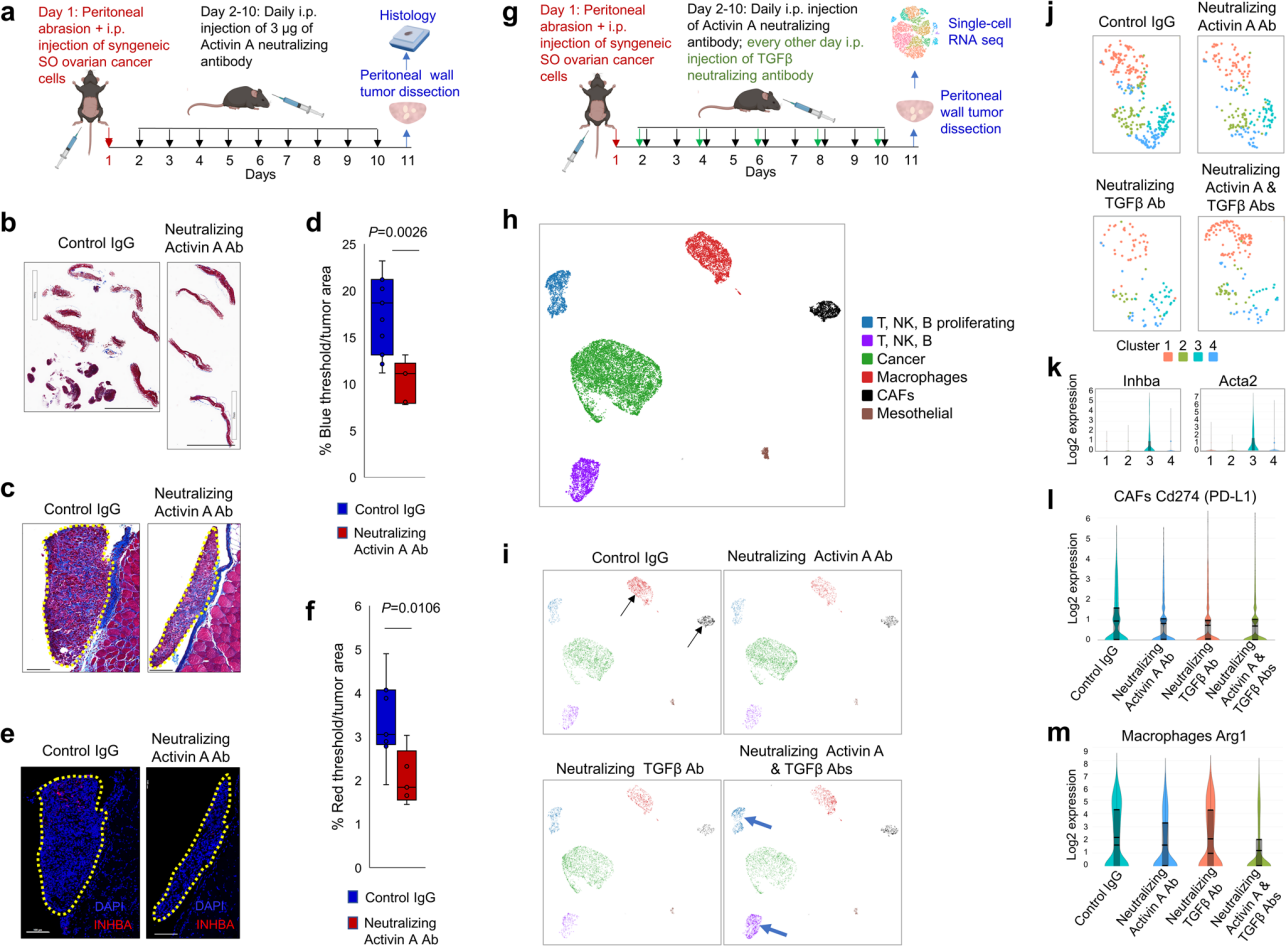
Activin A neutralizing antibody attenuates ovarian cancer growth and intratumoral recruitment of pro-tumorigenic fibroblasts and macrophages. **a** Study schematic of treatment of mice with control IgG or Activin A inhibitory antibodies. **b** Trichrome-stained sections of peritoneal walls and associated tumor nodules. Scale bars: 7 mm. **c**, **d** Representative images of tumor nodules (yellow dotted line) attached to the peritoneal mesothelium covering the abdominal wall muscle and connective tissue in sections stained by **(c)** trichrome and **d** Inhba ISH. Scale bars: 100 µm. **e**, **f** Quantification (ImageJ software, unpaired *t* test) of **e** blue pixels representing fibroblasts and **f** red pixels representing INHBA(+) cells. Y axes represent percent positive pixels per tumor area. Boxes indicate the 25th to 75th percentiles, whiskers indicate the minimum and maximum values, and vertical lines indicate the mean values. **g** Study schematic of treatment of mice with control IgG or neutralizing antibodies for Activin A and TGFβ individually or in combination. **h** Single-cell RNA sequencing analysis of peritoneal walls represented by a UMAP plot of single cells color-coded for their assigned major cell types based on canonical markers. **i** UMAP plots of main cell types in mice with different antibody treatments. **j** UMAP representation of four distinct clusters in CAFs and the distribution of clusters in mice with different treatments. Black arrows indicate CAFs and macrophages while blue arrows indicate immune cells. **k** Violin plot expression of the pro-tumorigenic myofibroblast markers Inhba and Acta2 in the four distinct clusters of CAFs. **l** Violin plot expression of PD-L1 in CAFs in different treatment groups. **m** Violin plot expression of the immunosuppressive macrophage marker Arg1 in different treatment groups.

To determine the impact of INHBA(+) CAFs on ovarian cancer progression in vivo, we used two syngeneic ovarian cancer models: the FVB BR-luc (genotype p53^−/−^; Brca1^−/−^; Myc; Akt)^36,37^ and the peritoneal abrasion-facilitated C57BL/6 syngeneic SO model (genotype p53^−/−^; Myc; Hras)^38^. In the BR-luc model^36,37^, FVB mice were implanted intraperitoneally (i.p.) with BR-luc cancer cells. After six days, initial tumor growth was confirmed by intravital luciferase imaging (IVIS) and the mice were randomized into two treatment groups to receive control shRNAs or Inhba shRNAs i.p. three times per week. Thirty days after the implantation of cancer cells, the tumor burden was quantitated by IVIS. Mice that had been injected i.p. with Inhba shRNAs had a significantly decreased luciferase signal in the peritoneal cavity compared to mice injected with control shRNAs (Supplementary Fig. 9a), suggesting that the reduction of Inhba expression in the host fibroblasts reduces the rate of tumor growth. Meaningful quantification of Inhba(+) fibroblasts by Inhba ISH was hampered by sparse and highly focal fibroblast infiltration (Supplementary Fig. 9b). To facilitate uniform recruitment of host fibroblasts to the tumor at a predefined metastatic site, we used the peritoneal wall abrasion-facilitated ovarian cancer model in which peritoneal wound healing facilitates the recruitment of cancer cells and fibroblasts into the abraded wall. C57BL/6 mice with peritoneal wall abrasion were injected i.p. with syngeneic SO ovarian cancer cells^38^. One day after cancer cell injection, the mice received daily i.p. injections of the Activin A neutralizing antibody (*n* = 8) or control IgG (*n* = 7) (Fig. 2a). After 9 days of treatment, mice were euthanized to assess cancer cell implantation to the wound site (Fig. 2a). Visible tumor nodules on the peritoneal walls were present in 7/8 of IgG-treated mice and 4/7 of Activin A neutralizing antibody-treated mice. Histological examinations revealed that tumors in mice treated with the Activin A neutralizing antibody displayed notable reductions in tumor size (Fig. 2b), collagen accumulation (Fig. 2c, d), and the presence of INHBA(+) fibroblasts (Fig. 2e, f).

In the second set of experiments (Fig. 2g), C57BL/6 mice with peritoneal wall abrasion were injected i.p. with syngeneic SO ovarian cancer cells^38^. One day after cancer cell injection, the mice received i.p. injections of control IgG, Activin A neutralizing antibody, TGFβ neutralizing antibody, and a combination of Activin A and TGFβ neutralizing antibodies (n = 5 mice per group). After 9 days of treatment, mice were euthanized and peritoneal walls with tumors were subjected to single cell RNA sequencing to identify individual cell types recruited to the site of cancer cell attachment in each group of mice. Cell clusters were identified using the 10X Genomics Loupe browser. Cell phenotypes were assigned based on the canonical cell-specific genes differentially expressed in each cluster (Fig. 2h)^39,40^. Uniform Manifold Approximation and Projection (UMAP) clustering analysis revealed that tumors from mice treated with the Activin A neutralizing antibody, TGFβ neutralizing antibody and a combination of both Activin A and TGFβ neutralizing antibodies had a reduced number of CAFs and macrophages compared to mice treated with control IgG antibody (Fig. 2i, black arrows). Additionally, mice treated with both Activin A and TGFβ neutralizing antibodies had an increased number of immune T, NK, and B cells compared to mice treated with IgG or individual neutralizing antibodies (Fig. 2i, blue arrows). Re-clustering of fibroblasts revealed four distinct clusters, of which cluster 3 expressed the highest levels of the pro-tumorigenic myofibroblast markers Inhba and Acta2 (Fig. 2j, k). This cluster of fibroblasts was moderately reduced in mice treated with Activin A inhibitory antibody and strongly reduced in mice treated with TGFβ inhibitory antibody and a combination of both antibodies (Fig. 2k). Programmed cell death ligand 1, PD-L1 (Cd274), was reduced in CAFs in all three treatment groups compared to control IgG mice (Fig. 2l). In re-clustered macrophages, levels of the pro-tumorigenic M2 macrophage marker arginase 1 (Arg1) was moderately reduced in mice treated with Activin A inhibitory antibody and drastically reduced in mice treated with the combination of the inhibitory antibodies (Fig. 2m).

### INHBA is required for CAF function in vitro

**Fig. 3:**
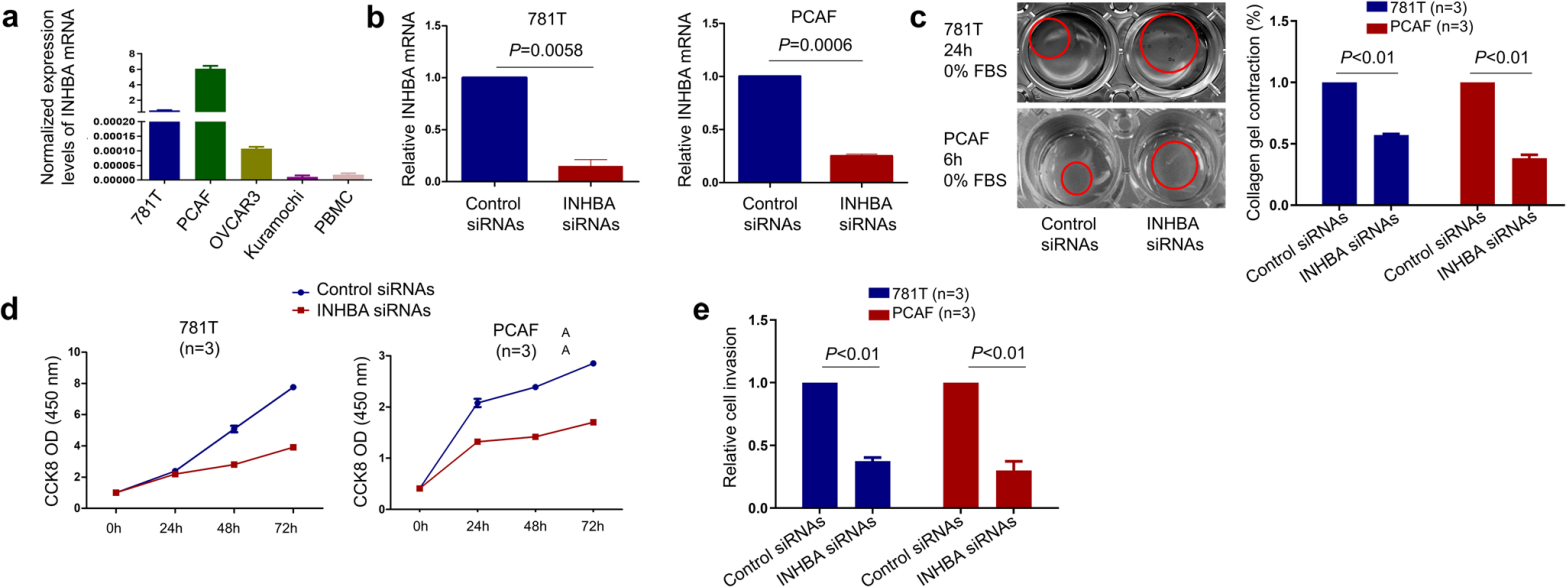
INHBA regulates CAF function in vitro. **a** INHBA levels measured by qRT-PCR in immortalized ovarian CAFs (781T), primary ovarian CAFs (PCAF), human ovarian cancer cell lines (OVCAR3 and Kuramochi), and PBMCs from a healthy donor. RPL32 was used for normalization. **b** qRT-PCR quantification of INHBA levels in two types of ovarian CAFs (immortalized 781T and primary PCAF) transduced with control SMARTpool siRNAs (control siRNAs) or INHBA SMARTpool siRNAs (INHBA siRNAs). INHBA levels were quantified 48 hours after treatment with siRNAs. RPL32 was used for normalization. **c** Representative image (left) and quantification (right) of collagen gel contraction assay with 781T CAFs and PCAFs transduced with control siRNAs or INHBA siRNAs. Red circles outline the perimeter of the collagen plug. The quantification was conducted 24 hours after plating 781T CAFs and 6 hours after plating PCAFs. **d** Proliferation of 781T CAFs and PCAFs transduced with control siRNAs or INHBA siRNAs. Cell growth rates were determined by the CCK-8 viability assay. The x-axis shows the time after transfection. The y-axis shows the readout of the CCK-8 assay (absorbance at 450 nm). **e** Transwell invasion assay with 781T CAFs and PCAFs transduced with control siRNAs or INHBA siRNAs. Cells that invaded the membrane were quantified in 5 high-power fields. All in vitro quantification data in this figure are presented as the mean ± SEM in 3 or more replicates.

Compared to fibroblasts in normal tissues, CAFs have increased contractile, proliferative, and migratory abilities^1–3^. These properties are thought to be driven primarily by TGFβ^31,41,42^; however INHBA is typically co-expressed with TGFβ in CAFs. To study the functional properties of INHBA(+) CAFs in vitro, we used the immortalized HGSOC CAF cell line, 781T, and the primary ovarian CAF, PCAF. qRT-PCR confirmed a higher expression of INHBA in 781T CAFs and PCAFs compared to the epithelial ovarian cancer cell lines OVCAR3 and Kuramochi and peripheral blood mononuclear cells (PBMCs) (Fig. 3a). The primary CAFs expressed higher levels of INHBA than the immortalized CAFs (Fig. 3a). To study the function of INHBA in vitro, 781T CAFs and PCAFs were transduced with control SMARTpool siRNAs (control siRNAs) or INHBA SMARTpool siRNAs (INHBA siRNAs). Downregulation of INHBA expression in 781 T CAFs and PCAFs was confirmed by qRT-PCR 48 hours after treatment (Fig. 3b). Downregulation of INHBA in both 781T CAFs and PCAFs resulted in a significant reduction of collagen gel contraction (Fig. 3c), cell proliferation (Fig. 3d), and cell invasion (Fig. 3e), indicating functional involvement of INHBA in these processes.

### INHBA(+) CAFs attract T cells and promote their differentiation into Tregs

**Fig. 4:**
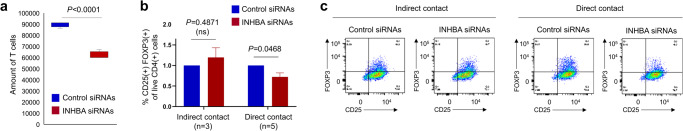
INHBA(+) CAFs attract T cells and facilitate their differentiation into Tregs via a contact-directed mechanism. **a** A Transwell migration assay of T cells toward 781T CAFs with or without downregulated INHBA expression. 781T CAFs treated with control siRNAs or INHBA siRNAs were plated in the bottom chamber of the Transwell plate (3.5 × 10^4^ cells/well). The following day, human CD3(+) pan T cells (5 × 10^6^) were plated in the upper chamber. After 36 hours of co-culture, T cells that migrated to the bottom chamber were collected and counted with a hemocytometer. The *P* value denotes student *t* test in triplicates. **c** Representative flow cytometry graphs of live CD4(+) T cells showing the frequency of CD25(+) FOXP3(+) Tregs after human pan T cells were activated with CD3/CD28 antibodies and co-cultured in direct contact (5 independent experiments) or indirect contact (Transwell membrane; 3 independent experiments) with 781T CAFs treated for 24 hours with control siRNAs or INHBA siRNAs. **b** Quantification of Tregs in co-cultures of activated human pan-T cells and 781T CAFs in direct and indirect contact (unpaired *t* test).

Multiple studies have demonstrated that CAFs are important regulators of anti-tumor immune responses^43–45^. To test whether expression of INHBA in CAFs is important for T cell attraction, we downregulated INHBA in 781T CAFs using INHBA siRNAs. 781T CAFs treated with INHBA siRNAs showed an impaired ability to attract T cells through the transwell membrane compared to 781T CAFs treated with control siRNAs (Fig. 4a). To determine the effect of CAFs on T cell proliferation and differentiation, we co-cultured 781T CAFs and human CD3(+) pan T cells in direct contact or indirect contact (cells separated by a transwell membrane). With direct contact, fewer CD4(+) CD25(+) FOXP3(+) Tregs were observed in the 781T CAFs with downregulated INHBA compared to the control while no difference in CD4(+) CD25(+) FOXP3(+) Tregs was observed with indirect contact (Fig. 4b, c). Based on these observations we conclude that INHBA(+) fibroblasts play a role in shaping the tumor immunosuppressive microenvironment by inducing expansion of the pro-tumorigenic CD4( +) CD25(+) FOXP3(+) Tregs.

### INHBA(+) CAFs induce an immunosuppressive tumor microenvironment by regulating PD-L1

In patients with metastatic urothelial bladder cancer treated with the anti-PD-L1 agent atezolizumab^4^, INHBA mRNA levels were higher in non-responders than in responders (Supplementary Fig. 10), suggesting that INHBA might be associated with immunosuppression. PD-L1 plays a key role in the differentiation of CD4(+) T cells into FOXP3-expressing Tregs^46,47^. CAFs have been shown to express PD-L1 and suppress proliferation of CD4(+) effector T cells via a contact-mediated mechanism^47–50^. Our analysis of the single-cell RNA-seq dataset in metastatic melanoma^51^ confirmed that CD274 (PD-L1) is expressed in CAFs (Supplementary Fig. 11a). Additionally, our analysis of a single-cell RNA-seq dataset of stromal cell diversity associated with immune evasion in human triple‐negative breast cancer^52^ in which CAFs that were separated into inflammatory CAFs (iCAFs) and myofibroblast-like CAFs (myCAFs) showed higher levels of both INHBA and CD274 in myCAFs than in iCAFs (Supplementary Fig. 11b).

**Fig. 5:**
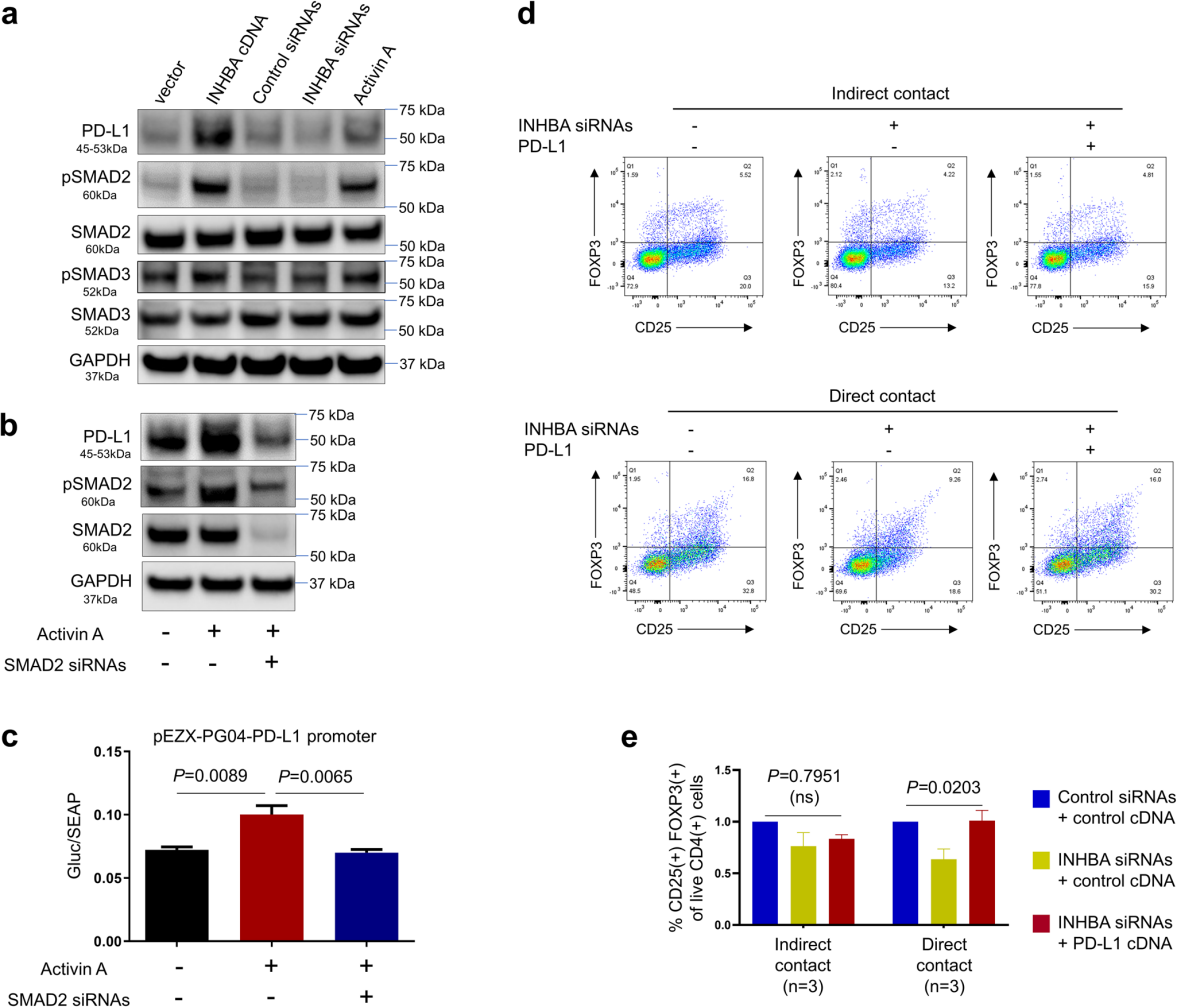
INHBA(+) CAFs create an immunosuppressive tumor microenvironment through the upregulation of PD-L1. **a** Western blot of PD-L1, phosphorylated SMAD2 (pSMAD2), SMAD2, pSMAD3, and SMAD3 in 781T CAFs were stably transfected with either INHBA cDNA/control cDNA, or transiently transfected with INHBA siRNAs/control siRNAs, or treated with recombinant Activin A. GAPDH was used as a loading control. INHBA mRNA levels measured by qRT-PCR in cell cultures grown in parallel with the cell cultures used for Western blotting are plotted in Supplementary Fig. 12. **b** Western blot detection of PD-L1, pSMAD2, SMAD2 in 781T CAFs were treated with negative controls, or Activin A and control siRNAs, or Activin A and SMAD2 siRNAs, followed by INHBA mRNA levels measured by qRT-PCR in cell cultures grown in parallel with the cell cultures used for Western blotting are plotted in Supplementary Fig. 12. **c** Measurements of GLuc and SEAP activities in the media using a GloMax microplate reader in 781T CAFs treated as described in **b**. Normalized promoter activity was calculated as the ratio of GLuc and SEAP activities (paired *t* test). **d** Representative flow cytometry graphs of live CD4(+) T cells showing the frequency of CD25(+) FOXP3(+) Tregs after human pan T cells were activated with CD3/CD28 antibodies and co-cultured in direct contact (3 independent experiments) or indirect contact (Transwell membrane; 3 independent experiments) with 781T CAFs treated for 24 hours with negative controls, or INHBA siRNAs and control cDNA, or INHBA siRNAs and PD-L1 cDNA. **e** Quantification of Tregs in co-cultures of activated human pan-T cells and 781T CAFs in direct and indirect contact (one-way ANOVA test).

781T CAFs expressed a moderate level of PD-L1 protein when grown in cell culture (Fig. 5a). Downregulation of INHBA by pooled siRNAs decreased the PD-L1 protein level, whereas ectopic expression of INHBA cDNA or addition of recombinant Activin A (Supplementary Fig. 12) increased PD-L1 protein levels (Fig. 5a). SMAD2 and SMAD3 are transcription factors associated with Activin A signaling^53^. Ectopic expression of INHBA cDNA and the addition of recombinant Activin A increased the expression of phosphorylated SMAD2 (pSMAD2) and, to a lesser extent, phosphorylated pSMAD3 (pSMAD3) (Fig. 5a and Supplementary Fig. 12). To explore the mechanism by which Activin A might regulate PD-L1 expression, we downregulated SMAD2 in 781T CAFs with a SMAD2-specific siRNA pool and measured the ability of recombinant Activin A to increase the levels of PD-L1. Recombinant Activin A increased levels of PD-L1 and pSMAD2 in 781 T CAFs treated with control siRNAs but this effect was abrogated by treatment with SMAD2 siRNAs (Fig. 5b and Supplementary Fig. 12), suggesting that Activin A increases PD-L1 levels through SMAD2 phosphorylation. The PD-L1 promoter region has several SMAD binding elements (CAGA box) (Supplementary Fig. 13). We tested whether Activin A can modulate the transactivation of PD-L1 mRNA. The promoter sequence of PD-L1 was cloned upstream of a luciferase reporter gene and the resultant construct was co-transfected with SMAD2 siRNAs or control siRNAs in 781T CAFs. Recombinant Activin A increased the transactivation of the PD-L1 promoter but this effect was abrogated by SMAD2 siRNAs (Fig. 5c), suggesting that Activin A promotes PD-L1 expression through SMAD2 dependent transactivation of the PD-L1 promoter. INHBA downregulation in 781T CAFs suppressed Treg expansion in a co-culture system, which was rescued by ectopic expression of PD-L1 (Fig. 5d, e), suggesting that INHBA confers PD-L1-dependent effects on CD4(+) Treg expansion.

## Discussion

Consistent with reports that INHBA is upregulated in multiple cancers including ovarian cancer^54^, we showed that INHBA expression in HGSOC is associated with poor patient survival. However, our study shows that the mechanism by which INHBA/Activin A contribute to ovarian cancer progression is different than previously thought. The main mechanism by which INHBA/Activin A contribute to ovarian cancer progression was thought to be the induction of epithelial-mesenchymal transition^18^. More recently, a new mechanism was proposed wherein INHBA-expressing cancer cells activate CAFs, which in turn promotes cancer progression^22,55^. Our ISH studies of primary, metastatic and recurrent human HGSOC samples demonstrated that CAFs, rather than epithelial cancer cells, are the main cell type expressing INHBA. It is likely that cancer cells are responsible for the induction of INHBA expression in CAFs as INHBA(+) CAFs were observed only in proximity to cancer cells. Additionally, our ISH analysis of tumor sections from syngeneic mouse models showed that INHBA is expressed in the host CAFs only if they are in proximity of cancer cells. The ratio of INHBA(+) CAFs in patients was higher in metastatic HGSOC tumors (synchronous and metachronous) compared to patient-matched primary tumors, implying either a phenotypic switch in fibroblasts imparted by the cancer cells during disease progression or recruitment of a distinct population of CAFs at the metastatic site compared to primary site.

Immunotherapies targeting CTLA-4, PD-1, and PD-L1 have demonstrated variable efficacy in solid malignancies^56^. Expression levels of the target genes and the presence of immune cells are not reliable predictors of response to immunotherapy^56^. Co-evolution of cancer cells and their microenvironment ultimately leads to immune tolerance and evasion, suggesting that targeting the tumor microenvironment could increase immunotherapeutic efficacy^57^. Studies in several cancer types have shown that the signature of TGFβ-mediated ECM remodeling is the best predictor of immunotherapy failure^8^ and that immunotherapeutic efficacy can be markedly improved by targeting CAFs^57^.

Nevertheless, indiscriminate targeting of tissue resident fibroblasts along with CAF populations has been counterproductive and, in some cases even induced cancer progression and caused significant toxicity due to the loss of normal fibroblasts that restrict tumor growth. For example, ablation or inactivation of CAFs using general myofibroblast markers, such as αSMA, FAP, and type-I collagen, induced severe toxicity and promoted cancer aggressiveness in mouse models^57^. Similarly, the success of combining TGFβ inhibitors with immunotherapy in animal cancer models could not be replicated in human clinical trials probably reflecting the pleiotropic functions of TGFβ in normal fibroblasts^9,58–60^ or a compensatory increase in Activin A signaling upon inhibition of TGFβ^15^. Thus, therapeutic targeting of the CAF/ECM/immune cell interface requires a better understanding of CAF diversity to develop more effective and less toxic therapies that selectively deplete only the tumor-promoting stromal components.

In this study, we showed that only a subset of αSMA(+) CAFs express INHBA. Unlike αSMA(+) CAFs, INHBA(+) CAFs were enriched in HGSOC metastases and their proportion correlated with poor patient survival, suggesting that INHBA(+) CAFs might be active participants in cancer progression. A feature that makes INHBA(+) CAFs a suitable therapeutic target is the low expression of INHBA in normal fibroblasts. As a TGFβ superfamily member, Activin A shares the canonical SMAD2/3 pathway with TGFβ and signals through its own set of receptors which are not utilized by TGFβ. Unlike TGF-β1, -β2, and -β3 homodimers which are secreted into ECM as inactive precursors and require proteolytic activation to exert their biological functions^42^, Activin A is secreted as an active dimer. As such, the mechanism for controlling Activin A signaling is different from the mechanisms that control the activation of TGFβ.

Considering the overlapping functions of Activin A and TGFβ, targeting Activin A might be an effective method to inhibit tumor progression without significant toxicity to normal tissues. A natural inhibitor of Activin A is Follistatin (FST)^21,61,62^. Activin A and FST are usually co-expressed in tissues, suggesting that FST is required for fine-tuning of Activin A signaling^53^. FST neutralizes Activin A by forming a non-signaling complex that prevents interaction with TGFβ receptors^62–64^. Overexpression of FST in a xenograft breast cancer model was shown to inhibit tumor growth^65^. Transgenic expression of FST in the autochthonous mouse HER2/Neu breast cancer model had no impact on tumor initiation or growth but it completely blocked the formation of lung metastases^66^. Similarly, in a mouse model of pancreatic cancer, treating mice with an Activin A neutralizing antibody did not reduce primary tumor size but it decreased tumor metastases^27^. Therefore, the treatment of patients with an Activin A neutralizing antibody or FST might be effective in metastasis suppression. However, producing large quantities of engineered recombinant human FST as anti-cancer therapy has proven difficult^67,68^. Garetosmab, a fully human monoclonal antibody that inhibits Activin A is under investigation for the treatment of fibrodysplasia ossificans progressiva (FOP) (NCT03188666)^69^ but has not been investigated in cancer patients. Although no Activin A inhibitor is currently available for clinical use in cancer patients, STM 434, a soluble receptor ligand trap targeting Activin A, is under clinical investigation for the treatment of refractory ovarian cancer (NCT02262455). Aside from the reduction in cachexia in some patients, data from this first-in-human phase I trial did not show clinical response^70^. Our study suggests that an Activin A blockade might be more effective as a component of a multipronged approach that includes immunotherapy. However, we acknowledge the nascent stage and the existing barriers in the clinical applications of Activin A targeting drugs.

PD-L1 levels are not always consistent with patient response rates to the PD-L1 checkpoint blockade, underscoring the need to better understand the underlying mechanism by which PD-L1 is regulated in different cell types in the tumor microenvironment. Our study showed that one of the mechanisms by which INHBA(+) CAFs can promote cancer progression is autocrine upregulation of PD-L1. We showed in human CAFs that INHBA transactivates PD-L1 promoter activity through pSMAD2. In co-culture studies, we showed that INHBA(+) CAFs are able to recruit CD3(+) T cells and promote their differentiation into Tregs. Consistent with in vitro co-culture data, a high Treg cell ratio was observed in the tumor microenvironment of patients with a high INHBA(+) CAF ratio, which was associated with shorter overall survival. The induction of Tregs by Activin A has been described in asthma^12,13^ and the 4T1 syngeneic mouse model of breast cancer^15^ but the mechanism by which Activin A induces Treg differentiation was unknown. In this study, we showed in human ovarian CAFs that Treg differentiation is dependent on direct contact with INHBA(+) CAFs in which Activin A induces phosphorylation of SMAD2.

## Methods

### Patient samples, TMA, and gene expression analyses by RNA sequencing

The studies using human samples complied with all relevant ethical regulations including the Declaration of Helsinki. Collection of archival human samples and procedures using the human samples were approved by the Cedars-Sinai Medical Center Institutional Review Board (IRB) or the University of California Los Angeles IRB. All patients provided written consent for use of their tissues for research aimed at advancing the understanding of ovarian cancer. This includes the dissemination of research results through publications. Formalin-fixed paraffin-embedded blocks were retrieved from the pathology archives at Cedars-Sinai Medical Center. The TMA of patient-matched primary ovarian cancer, synchronous pre-treatment metastasis, and metachronous post-treatment recurrence samples from 42 patients with HGSOC was generated. Each primary, metastatic, and recurrent HGSOC sample was represented by triplicate 1 mm cores that were punched at different locations in the corresponding original FFPE tumor block. The 378 cores were distributed on two slides. All of the patients had primary debulking surgery followed by 3-6 cycles of platinum/taxane-based chemotherapy. For the GSE138866 data set, samples of omental metastases collected from 130 HGSOC patients at the time of primary debulking surgery were analyzed for RNA expression by RNA sequencing using the SMARTer Stranded Total RNA-Seq Kit v2 on the Illumina HiSeqX platform (MedGenome). Unwanted sequences (non-polyA tailed RNAs from the sample, mitochondrial genome sequences, ribosomal RNAs, transfer RNAs, adapter sequences and others) were removed using Bowtie2 (version 2.2.4). The paired-end reads were aligned to the reference human genome downloaded from the UCSC database (GRCh37/hg19). STAR (2.4.1) aligner was used for read alignment. Reads mapping to ribosomal and mitochondrial genomes were removed before alignment was performed. The raw read counts were estimated using HTSeq-0.6.1. Read count data were normalized using DESeq2.

### ShRNA-mediated INHBA downregulation in a syngeneic mouse ovarian cancer model

All procedures in mice in this experiment were performed in accordance with the NIH Guide for the Care and Use of Laboratory Animals and approved by the Institutional Animal Care and Use Committee (IACUC) of Cedars-Sinai Medical Center. Six to eight week-old female FVB mice (The Jackson Laboratory, Sacramento, CA, USA) were implanted with 1 × 10^6^ of syngeneic BR-Luc mouse ovarian cancer cells (genotype: p53^−/−^, Brca1^−/−^, myc, Akt)^36,37^. Six days after cancer cell implantation, the mice were imaged using the IVIS Spectrum CT In Vivo Imaging System (Perkin Elmer Inc., Waltham, MA, USA) to confirm tumor growth. On the same day, either control shRNA scrambled for psi-U6 (CSHCTR001-CU6, GeneCopoeia) or INHBA shRNA (MSH093015-CU6, GeneCopoeia) was injected i.p. (100 µg shRNA per mouse) and continued to be injected three times per week for 30 days. The shRNA was prepared for injection by mixing with 16 µl of the lipophilic transfection reagent (in vivo-jetPEI; Polyplus-transfection, New York, USA) in 5% glucose solution according to the reagent protocol. Thirty days after cancer cell implantation, the mice were subjected to IVIS imaging to quantify tumor burden. Mice were euthanized by CO_2_ asphyxiation followed by cervical dislocation. Tumor tissue was harvested and fixed in 10% formalin and processed for paraffin embedding.

### Activin A and TGFβ neutralizing antibody-mediated inhibition of Activin A and TGFβ signaling, individually and in combination, in a syngeneic mouse model of peritoneal abrasion-facilitated ovarian cancer

All procedures in mice in this experiment were performed in accordance with the NIH Guide for the Care and Use of Laboratory Animals and approved by the Animal Research Committee (ARC) of the University of California Los Angeles. Six- to eight-week-old female C57BL/6 mice (Charles River Laboratories) were subjected to minimally-invasive surgical abrasion of the abdominal peritoneal wall. The surgery was performed by inserting a dental microblade through the skin on the ventral side and performing an abrasion of the peritoneal wall. After surgery, mice were intraperitoneally injected with 0.5×10^6^ of syngeneic SO mouse ovarian cancer cells^38^. The following day, mice were randomized into four groups that received daily i.p. injections of control mouse IgG (mABb002, R&D Systems), 3 µg of Activin A beta A subunit neutralizing antibody (mAb3381, R&D Systems), and/or 3 mg/kg of TGFβ neutralizing antibody (clone 1D11.16.8., BioXCell) every other day. After 9 days of treatment, mice in both groups were euthanized by CO_2_ inhalation followed by cervical dislocation. From each mouse, 1-3 mm^3^ pieces of abdominal wall tumors at the site of peritoneal abrasion were harvested, combined into one sample per group, and prepared for immunohistochemical analysis or single-cell RNA sequencing.

### Single-cell RNA sequencing

Tumors collected from the site of peritoneal abrasion from the mice treated with Activin A neutralizing antibody or control IgG were pooled and minced into 1 mm pieces. Tissue was further dissociated by enzymatic digestion by rotating at 37 °C for 30 minutes with 1 mg/ml Collagenase/Hyaluronidase (STEMCELL), DNAse, and trypsin (0.01%). After dissociation, the suspension was strained (70 µm) followed by centrifugation (8 minutes at 300 g). Red blood cells were removed using the red blood cell lysis buffer (Thermo Fisher) followed by dead cell removal (STEMCELL) according to the manufacturer’s instructions. Finally, cells were resuspended in 0.04% BSA-PBS solution at a concentration of 700-1000 cells/microliter without selection for specific cell types. Single-cell RNA sequencing was performed by the UCLA David Geffen School of Medicine Technology Center for Genomics & Bioinformatics Core. Cell numbers in samples were quantified using the Countess II Automated Cell Counter (Thermo Fisher Scientific). Single-cell gene expression libraries were created according to the manufacturer’s instructions using the following reagents from 10X Genomics: Chromium Next GEM Single-Cell 3’ (v3.1 Chemistry), Chromium Next GEM Chip G Single Cell Kit, and Single Index Kit TT Set A. Cells were loaded to target 10 K cells per sample to form GEMs and barcode individual cells. cDNA and libraries were created according to the manufacturer’s instructions. Library quality was assessed using the 4200 TapeStation System and D1000 ScreenTape (Agilent) and Qubit 2.0 (Invitrogen) for concentration and size distribution. Samples were sequenced using the NovaSeq6000 SP (Illumina) with 100 cycles (28 + 10 + 10 + 91). 200 M reads were targeted for each sample, targeting 20 K reads per cell. The 10xGenomics CellRanger software suite version 7.0.1 was used to perform sample demultiplexing, alignment, barcode processing, and unique molecular identifier (UMI) quantification. The 10xGenomics Loupe Browser version 6.3.0 was used for thresholding and data analysis. Cells were removed according to the following criteria: 1) cells had fewer than 1 K genes or more than 8.5 K genes; 2) cells had fewer than 1 K unique molecular identifier (UMI) or over 100 K UMI; and 3) cells had more than 10% of mitochondrial UMI counts.

### Bright light image data analysis

In situ hybridization (ISH) with human Hs-INHBA probe (RNAScope, Cat# 415111) and mouse Mm-Inhba probe (RNAScope, Cat# 4155871) was performed using protocols provided by RNAScope. Immunohistochemistry (IHC) with the α-SMA antibody (α-am-1, Leica Biosystems, manufacturer prediluted to working concentration for autostainer) was performed by the Cedars-Sinai Medical Center Biobank and Translational Research Core using standard protocols for automated immunostaining. The stained slides were scanned at 20x magnification using Aperio AT Turbo. The image analysis was performed using the QuPath software. The image analysis workflow consisted of cell/nucleus detection, annotation of the regions containing four different cell types (fibroblast, epithelial cancer cell, immune cell, and others), creation of the cell detection classifier, and application of the classifier to all cells in the circled regions of the slide. DAB staining intensity classification was applied to annotate positive/negative cells. The cores with <500 cancer cells were excluded and the remaining cores were averaged for further analysis.

### Multiplex immunofluorescence assay and data analysis

Multiplex immunofluorescence assay was performed by the Cedars-Sinai Medical Center Biobank and Translational Research Core using experimental protocols and data analysis software that has been previously reported^71^. All antibodies were prediluted by the Cedars-Sinai Medical Center Biobank and Translational Research Core to appropriate dilutions for autostainer or purchased prediluted from the manufacturer.CD8 (clone JCB117, Ventana), CD3 (clone 2GV6, Ventana), CD4 (clone SPO32, Cell Marque), and FOXP3 (clone 236 A/E7, ThermoFisher Scientific) antibodies were used to identify T cells while α-SMA (clone α-am-1, Leica Biosystems) and cytokeratin 8/18 (clones B22.1/B23.1, Ventana) antibodies were used to identify fibroblasts and epithelial cells, respectively. DAPI was used to outline nuclei. Slides were imaged with a TissueFAX whole slide scanning platform (TissueGnostics USA Ltd.) equipped with a 20x objective and a scientific-grade 16-bit monochromatic camera (1392 × 1040 pixels). The gray scale images of the IF images were thresholded using Matlab software. Thresholds were visually adjusted using images from different cores. After thresholding, a binary image was created for each channel and image tile and positive pixels were quantified. Pixel numbers were exported together with the area from which they were obtained. Pixel groups with fewer than 9 pixels were excluded from the analysis. A nuclear segmentation algorithm was applied to DAPI images to generate a nuclear mask. The nuclear outline was expanded into a doughnut by a fixed length equal to 1/3 of the mean nuclear radius. The cell was classified as positive if the positive pixel density within the doughnut exceeded a pre-defined threshold (CD3:240, CD4:640, CD8:520, and FOXP3:1200). The process was repeated for all antibody channels. Binary masks from the pixel-based segmentation approach were used to analyze T cell populations. Single positive pixels (pixels colored only by one of the antibodies) were counted after excluding double and higher order labeled pixels from individual antibody masks. Double positive pixels (pixels positive for two antibodies) were generated by the intersection of two masks. Triple positive pixels (pixels positive for three or more antibodies) were identified by the overlap of pixels of three masks. Binary masks of cancer cells and fibroblasts were generated from unmixed images of mIF slides. The masks for cancer cells and fibroblasts were obtained by identifying Keratin 8/18 and α-SMA positive cells, respectively. For example, binary masks were obtained with Keratin 8/18 in the foreground (cancer cells mask, white pixels) and the remaining tissue components in the background (black pixels). Subtypes of CD3(+), CD4(+), and FOXP3(+) immunocytes in cancer cell and fibroblast masks were analyzed using GraphPad PRISM (version 8.0; GraphPad Software).

### Cell culture

A human ovarian primary cancer associated fibroblast (PCAF) was generated from a discarded tissue from an HGSOC patient who underwent primary surgical tumor resection. The discarded tissue was provided by the Pathology Core at Cedars-Sinai Medical Center after patient’s written consent for use of discarded tissue for research aimed at advancing the understanding of ovarian cancer. This includes the dissemination of research results through publications. The studies using human samples complied with all relevant ethical regulations including the Declaration of Helsinki. The tumor tissue was manually minced with a sterile scalpel and enzymatically digested by rotating at 37 °C for 30 minutes with 1 mg/ml Collagenase/Dispase (Roche). The cells were isolated by filtering the tissue through an 80 µm filter, centrifugation at 400 g for 8 minutes, lysing red blood cells (8 g NH4Cl, 1 g KHC03 in 1 L H2O, pH adjusted to 7.2), and another centrifugation at 400 *g* for 5 minutes. The cell pellet was washed with a wash buffer twice and filtered through an 80 µm filter, followed by additional filtration through a 40 µm filter. The cells were seeded in a 10 cm dish and cultured at 37 °C in 5% CO_2_ until confluent. Flow cytometry analysis was conducted to confirm that the cells were CD45(-) CD31(-) CD326 (-) using PE/Cy7 anti-human CD31, BioLegend #303118, 5 µl in 100 µl staining volume; Alexa Fluor 647 anti-human CD326, Biolegend #324212, 5 µl in 100 µl staining volume; Brilliant Violet 421™ anti-human CD45, BioLegend #304032, 5 µl in 100 µl staining volume. 781 T CAFs were obtained from Kate Lawrenson at Cedars-Sinai Medical Center and immortalized with hTERT to generate 781T CAFs. PCAF and 781 T CAFs were maintained in McCoy’s 5A tissue culture medium (Corning) supplemented with 10% fetal bovine serum (FBS) and 1% penicillin-streptomycin. The FVB mouse syngeneic ovarian cancer cell line BR-luc (genotype BRCA1^−/−^, Trp53^−/−^, Myc, Akt) has been described^36,37^ The C57BL/6 mouse syngeneic ovarian cancer cell line SO (genotype Trp53^−/−^, Myc, Hras) was generated by isolating ovaries from p53^−/−^ mice and transfecting them in vitro with pWzl-hygro-H-Ras V12 (#18749) and pWzl-blast-Myc (#10674) as previously described^38^. Human ovarian cancer cell lines Kuramochi and OVCAR3 were maintained in DMEM (Corning) supplemented with 10% FBS and 1% penicillin-streptomycin. The authenticity of the 781T CAFs and Kuramochi ovarian cancer cells was confirmed by Applied Biosystems. The authenticity of OVCAR3 cells was confirmed by Laragen. Human T cells were cultured in RPMI 1640 (Corning) supplemented with 10% FBS and 1% penicillin-streptomycin. All cells were cultured at 37 °C in 5% CO_2_.

### RNA isolation and quantitative real-time PCR (qRT-PCR) analysis

Total RNA was extracted using the RNeasy mini kit (Qiagen) and was reverse-transcribed to cDNA using the QuantiTect Reverse Transcription Kit (Qiagen). cDNA was then mixed with primers and iQ SYBR Green Supermix (#1708882, Bio-Rad) in a 96-well plate format. The qRT-PCR reaction was performed using an iCycler thermo cycler (Bio-Rad) and the data were analyzed by the Embedded Image method. Primers for human INHBA (Forward: ACGGGTATGTGGAGATAGAGG, Reverse: TGGAAATCTCGAAGTGCAGC) and ribosomal protein L32 (RPL32) (Forward: 5’-ACAAAGCACATGCTGCCCAGTG-3’; Reverse: 5’-TTCCACGATGGCTTTGCGGTTC-3’) were purchased from Invitrogen. RPL32 served as an internal control. 2^−Δct^ values were determined for analysis of relative gene expression. Expression levels of INHBA were plotted and analyzed by an unpaired *t*-test using GraphPad PRISM (version 8.0; GraphPad Software).

### Target downregulation by siRNA

For siRNAs experiments, 781T CAFs were seeded in a 6-well plate (1.3 x 10^5^ cells per well). The cells were cultured in McCoy’s 5A medium with 10% FBS, which was replaced the following day with McCoy’s 5A medium with 2% FBS for the siRNAs transfection. SMARTpool: ON-TARGETplus Human INHBA siRNA (L-011701-00-0005, Dharmacon) and SMARTpool: ON-TARGETplus SMAD2 siRNA (L-003561-00-0005, Dharmacon) were used to downregulate INHBA and SMAD2, respectively. ON-TARGETplus Non-targeting pool (D-001810-10-05, Dharmacon) was used as a negative control. siRNAs transfection was conducted according to the Lipofectamine RNAiMAX (Invitrogen #13778075) protocol. 781T CAFs were harvested after 24–48 hours for RNA isolation and after 48–72 hours for protein extraction.

### Treatment of cells with INHBA cDNA and recombinant Activin A protein

A vector expressing human INHBA cDNA (#RC203226, OriGene) and control cDNA (#PS100001, OriGene) were introduced into CAFs using the lipofectamine 3000 reagent (#L3000015, Thermo Fisher Scientific) according to the transfection protocol. CAFs were cultured in McCoy’s 5A with 1% FBS for 24 hours before treatment with 20 ng/ml of recombinant human Activin A (#338-AC-010, R&D Systems) for 24–48 hours.

### Collagen gel contraction assay

781T CAFs and PCAFs treated with control or INHBA siRNAs were mixed with rat tail collagen I (Advanced Biomatrix) then added to the 1% bovine serum albumin (BSA) pre-treated culture wells. The gels were polymerized for 1 hour at 37 °C and 5% CO2 before adding 600 µl of cell culture medium to each well. Collagen gel contraction was monitored over a period of 24 hours. To obtain gel contraction values, the relative diameters of the well and gel were measured using ImageJ (Fiji) software. The result of gel contraction was calculated using the formula: (well diameter-gel diameter)/well diameter. A detailed protocol for the gel contraction assay has been previously reported^38^.

### Cell viability assay

Cell viability was assessed by the Cell Counting Kit 8 (CCK-8; #CK04-05, Dojindo). Control and treated CAFs were seeded into 96-well plates at an initial density of 3000 cells/well. At each time point, 10 µl of CCK-8 solution was added to each well and incubated for 2 hours. The absorbance was measured by scanning with a microplate reader at 450 nm.

### Matrigel invasion assay

Matrigel invasion assays were performed using Boyden chambers with filter inserts (pore size, 8-μm) coated with Matrigel in 24-well dishes (BD Biosciences). 1 × 10^5^ to 2 × 10^5^ CAFs transfected with INHBA siRNAs or control siRNAs were seeded in the upper chamber, while McCoy’s 5A medium (Corning) supplemented with 30% fetal bovine serum was placed in the lower chamber. The plates were incubated for 24–48 hours. The cells were then fixed in 4% formaldehyde and stained with 0.05% crystal violet in PBS for 20 minutes at room temperature. Cells on the upper side of the filters were removed by cotton-tipped swabs and the filters were washed with PBS. The cells on the lower side of the filters were defined as invaded cells and quantified under the microscope in 5 high-power fields.

### Flow cytometry analysis

All cells were incubated with Live/Dead Fixable Blue stain (#L23105, Thermo Fisher Scientific) for 15 minutes at 4 °C. Surface antibody staining (anti-human CD4, CD8, CD25) was performed for 30 minutes at 4 °C, followed by fixation/permeabilization for 30 minutes at 4 °C (FOXP3/Transcription Factor Staining Buffer Set Kit, #00-5523-00, eBioscience). Cells were then stained for intracellular factor FOXP3 for 30 minutes at room temperature. The following antibodies were used at a concentration of 5 µl in 100 µl staining volume: CD8-Alexa Fluor 700 (#344724, BioLegend), CD4-APC/Fire 750 (#300560, BioLegendCD25-BV510 (#302639, BioLegend), and FOXP3-APC (#17-4777-42, eBioscience). All samples were acquired with an LSRII flow cytometer and analyzed with FlowJo software (version 10.4.0) and a multistep gating strategy to identify immune cells (Supplementary Fig. 14 and Supplementary Fig. 15).

### T cell isolation and co-culture experiments with CAFs

Pan T cells were isolated from the peripheral blood of healthy donors with the EasySep™ Human T Cell Enrichment Kit (#19051, StemCell) according to the manufacturer’s protocol. The patients provided written consent for use of their tissue for research, including the dissemination of research results through publications. Leukocyte reduction system chambers (LRSC) containing processed blood contents collected from healthy donors were washed with 2% FBS in PBS and collected into 50 ml conical tubes. The human peripheral blood mononuclear cell (PBMC) suspension was obtained from the LRSC contents by centrifugation over a density gradient medium (#07851, StemCell) using SepMate™ tubes (#85450, StemCell). Antibody complexes and magnetic particles were then added to the PBMC suspension. The magnetically labeled cells were separated from the unlabeled desired cells by using an EasySep™ magnet and pouring the desired cells into a new tube by immunomagnetic negative selection. Cell populations were >95% pure as confirmed by FACS analysis. For the T cell migration assay, 1.5 x 10^5^ human pan T cells were plated in the upper chamber of a Transwell-96 Permeable Support (#3388, Corning) while 1 x 10^4^ 781T CAFs treated with INHBA siRNAs or control siRNAs were plated in the bottom chamber for 24–36 hours. T cell migration was quantified by collecting T cells in the lower chamber and counting them with a hemocytometer. The number of T cells was calculated by cell concentration in the 200 µl solution in the bottom chamber. For the T cell differentiation assay, CAFs (1 × 10^5^/mL) were added to the upper Transwell chamber (#353495, Corning) and purified human pan T cells (1 × 10^6^/ml) were added to the lower chamber. CD3/CD28 T Cell Activator (#10991, StemCell) was added to the lower chamber. After five days of co-culture, T cells were harvested and analyzed for expression of FOXP3 and CD25 in live CD4+ T cells by flow cytometry.

### Western blotting

Whole cell lysates were prepared using RIPA buffer (Thermo Fisher) containing a protease and phosphatase inhibitor cocktail (Roche). After electrophoresis, proteins were electroeluted at 120 volts onto a polyvinylidene difluoride (PVDF) membrane (Invitrogen). The following antibodies (all from Cell Signaling Technologies) were used for Western blot analyses: PD-L1 (#13684, 1:1000 dilution), SMAD2 (#5339, 1:1000 dilution), pSMAD2 (#3104, 1:1000 dilution), SMAD3 (#9523, 1:1000 dilution), and pSMAD3 (#9520, 1:1000 dilution) and GAPDH (#5174, 1:1000 dilution). The Western blot analyses were replicated at least three times. All blots were derived from the same experiment and processed in parallel. Uncropped blots are provided (Supplementary Fig. 16).

### PD-L1 promoter luciferase reporter assay

The PD-L1-Gaussia Luciferase GLuc-ON promoter clone containing a 1282-base fragment upstream of the transcription start site and a 237-base fragment of the exon 1 of human PD-L1 in pEZX-PG04 vector (also containing a constitutively expressed secreted alkaline phosphatase (SEAP) secondary reporter as an internal control) was obtained from GeneCopoeia. PCAFs were plated in 24-well plates and maintained in McCoy’s 5 A media with 10% FBS and 1% penicillin-streptomycin at 37 °C in 5% CO_2_. Upon 80–90% confluence, cells were transfected with the following expression plasmids: 1 µg PD-L1-luc with various combinations of the following treatments: SMAD2 siRNAs, control siRNAs (mock transfection), and recombinant Activin A using the Lipofectamine 3000 transfection reagent (Thermo Fisher Scientific) according to the manufacturer’s protocol. Media were changed the following day and collected for measurement 48 hours after transfection. The secreted GLuc and SEAP activities were measured with the Secrete-Pair Dual Luminescence Assay Kit (#LF031, GeneCopoeia) according to the manufacturer’s protocol using a GloMax microplate reader (Promega). Normalized promoter activity was calculated as the ratio of GLuc and SEAP activities.

### Reporting summary

Further information on research design is available in the Nature Research Reporting Summary linked to this article.

### Supplementary information

REPORTING SUMMARY

Supplementary Figures 1-16

## Data Availability

Raw and normalized bulk and single-cell RNA sequencing data generated in this study were deposited into the gene expression omnibus (GEO) archive under the accession numbers GSE138866 and GSE229529, respectively. All other data, including immunohistochemistry and cell morphology analyses, survival analyses, and flow cytometry analyses will be made available upon request to the corresponding author, Dr. Sandra Orsulic, at SOrsulic@mednet.ucla.edu.

## Code availability

Codes were not generated for this study.
