# Supplementary material for: INHBA(+) cancer-associated fibroblasts generate an immunosuppressive tumor microenvironment in ovarian cancer
Source: NPJ Precis Oncol. 2024 Feb 15;8:35. doi: 10.1038/s41698-024-00523-y (PMC10869703; doi:10.1038/s41698-024-00523-y)
Supplement: Supplementary file 1 — REPORTING SUMMARY [file 41698_2024_523_MOESM1_ESM.pdf]

nature portfolio

Corresponding author(s): Sandra Orsulic

Last updated by author(s): Nov 14, 2023

Reporting Summary

Nature Portfolio wishes to improve the reproducibility of the work that we publish. This form provides structure for consistency and transparency in reporting. For further information on Nature Portfolio policies, see our Editorial Policies and the Editorial Policy Checklist.

Statistics

For all statistical analyses, confirm that the following items are present in the figure legend, table legend, main text, or Methods section.

n/a	Confirmed	
☐	☒	The exact sample size (*n*) for each experimental group/condition, given as a discrete number and unit of measurement
☐	☒	A statement on whether measurements were taken from distinct samples or whether the same sample was measured repeatedly
☐	☒	The statistical test(s) used AND whether they are one- or two-sided<br>*Only common tests should be described solely by name; describe more complex techniques in the Methods section.*
☐	☒	A description of all covariates tested
☐	☒	A description of any assumptions or corrections, such as tests of normality and adjustment for multiple comparisons
☐	☒	A full description of the statistical parameters including central tendency (e.g. means) or other basic estimates (e.g. regression coefficient) AND variation (e.g. standard deviation) or associated estimates of uncertainty (e.g. confidence intervals)
☐	☒	For null hypothesis testing, the test statistic (e.g. *F*, *t*, *r*) with confidence intervals, effect sizes, degrees of freedom and *P* value noted<br>*Give P values as exact values whenever suitable.*
☒	☐	For Bayesian analysis, information on the choice of priors and Markov chain Monte Carlo settings
☒	☐	For hierarchical and complex designs, identification of the appropriate level for tests and full reporting of outcomes
☐	☒	Estimates of effect sizes (e.g. Cohen's *d*, Pearson's *r*), indicating how they were calculated

Our web collection on statistics for biologists contains articles on many of the points above.

Software and code

Policy information about availability of computer code

Data collection	As shown in figures and labels in the manuscript and supplementary data
Data analysis	10xGenomics CellRanger software suite version 7.0.1 was used to perform sample demultiplexing, alignment, barcode processing, and unique molecular identifier (UMI) quantification. The 10xGenomics Loupe Browser version 6.3.0 was used for thresholding and data analysis.<br>For the GSE138866 data set, samples of omental metastases collected from 130 HGSOC patients at the time of primary debulking surgery were analyzed for RNA expression by RNA sequencing using the SMARTer Stranded Total RNA-Seq Kit v2 on the Illumina HiSeqX platform (MedGenome). Unwanted sequences (non-polyA tailed RNAs from the sample, mitochondrial genome sequences, ribosomal RNAs, transfer RNAs, adapter sequences and others) were removed using Bowtie2 (version 2.2.4). The paired-end reads were aligned to the reference human genome downloaded from the UCSC database (GRCh37/hg19). STAR (2.4.1) aligner was used for read alignment. Reads mapping to ribosomal and mitochondrial genomes were removed before alignment was performed. The raw read counts were estimated using HTSeq-0.6.1. Read count data were normalized using DESeq2.<br>Image analysis (bright field): QuPath<br>Image analysis (mIF): Akoya<br>IF Slides were imaged with a TissueFAX whole slide scanning platform (TissueGnostics USA Ltd.) The gray scale images of the immunofluorescent images were thresholded using Matlab software

For manuscripts utilizing custom algorithms or software that are central to the research but not yet described in published literature, software must be made available to editors and reviewers. We strongly encourage code deposition in a community repository (e.g. GitHub). See the Nature Portfolio guidelines for submitting code & software for further information.

Data

Policy information about availability of data

All manuscripts must include a data availability statement. This statement should provide the following information, where applicable:

- Accession codes, unique identifiers, or web links for publicly available datasets
- A description of any restrictions on data availability
- For clinical datasets or third party data, please ensure that the statement adheres to our policy

Raw and normalized bulk and single-cell RNA sequencing data generated in this study were deposited into the gene expression omnibus (GEO) archive under the accession numbers GSE138866 and GSE229529, respectively. All other data, including immunohistochemistry and cell morphology analyses, survival analyses, and flow cytometry analyses will be made available upon request to the corresponding author, Dr. Sandra Orsulic, at SOrsulic@mednet.ucla.edu.

Research involving human participants, their data, or biological material

Policy information about studies with human participants or human data. See also policy information about sex, gender (identity/presentation), and sexual orientation and race, ethnicity and racism.

Reporting on sex and gender	Ovarian cancer occurs only in females; males (human or mice) were not used in this study.
Reporting on race, ethnicity, or other socially relevant groupings	3 African American, 3 Asian, 36 Caucasian
Population characteristics	The average age at diagnosis was 55.4 (range 45.3- 62.1) years. Four patients had a prior history of cancer (two breast, one uterine, and one leukemia). The median disease-free interval (DFI) was 19.6 (range 5.5-51.7) months. Median overall survival (OS) was 65.9 (range 15.8-156.5) months. Four of 42 patients were alive at last follow-up (79.6, 91.2, 150.3, and 156.5 months).
Recruitment	For the ovarian cancer tissue microarray, archival FFPE samples from female patients ages 40-70 with high-grade serous ovarian cancer that were optimally debulked, then undergone 3-6 cycles of platinum/taxane-based therapy, then relapsed and required secondary surgery. All the samples included in this study were obtained from patients who granted their written consent for the utilization of their tissue for research purposes. Three types of tumors were collected from 42 patients – primary, synchronous metastatic, and metachronous recurrent tumors. Primary and synchronous metastatic tumors were acquired during primary debulking surgery (pre-chemotherapy) while metachronous/recurrent metastases were acquired during second-look surgery (post-chemotherapy). Primary ovarian tumors were collected from sites including the ovary, fallopian tube, or peritoneum, and synchronous and metachronous/recurrent metastases were collected from various intraperitoneal sites including the omentum, gastrointestinal organs, peritoneum or lymph node. A histologic diagnosis of HGSOC was confirmed in all tumor samples by pathology. After recovery from primary debulking surgery all patients were treated with 3-6 cycles of platinum-based chemotherapy. The study was conducted according to an approved protocol and in in accordance with the Declaration of Helsinki and the Good Clinical Practice guidelines.
Ethics oversight	Cedars-Sinai Medical Center (IRB and IACUC) and University of California Los Angeles (ARC)

Note that full information on the approval of the study protocol must also be provided in the manuscript.

Field-specific reporting

Please select the one below that is the best fit for your research. If you are not sure, read the appropriate sections before making your selection.

☒ Life sciences ☐ Behavioural & social sciences ☐ Ecological, evolutionary & environmental sciences

For a reference copy of the document with all sections, see nature.com/documents/nr-reporting-summary-flat.pdf

Life sciences study design

All studies must disclose on these points even when the disclosure is negative.

Sample size	For mice, sample size was calculated according to https://sample-size.net/all-378 samplescalculators-on-this-site/.<br>For the tissue microarray (TMA), the number of samples was set by the Cedars-Sinai Pathology Core that constructed the TMA (42 patients, 378 sample cores).
Data exclusions	For single-cell RNA sequencing, cells were removed according to the following criteria: 1) cells had fewer than 500 1K genes or more than 10000 8.5K genes; 2) cells had fewer than 500 1K unique molecular identifier (UMI) or over 10000 100K UMI; and 3) cells had more than 1510% of mitochondrial UMI counts. For IF, slides were imaged with a TissueFAX whole slide scanning platform (TissueGnostics USA Ltd.) equipped with a 20x objective and a scientific-grade 16-bit monochromatic camera (1392 × 1040 pixels). The gray scale images of the IF images were thresholded using Matlab software. Thresholds were visually adjusted using images from different cores. After thresholding, a binary image was created for each channel and image tile and positive pixels were quantified. Pixel numbers were exported together with the area from which they were obtained. Pixel groups with fewer than 9 pixels were excluded from the analysis. A nuclear segmentation algorithm

	was applied to DAPI images to generate a nuclear mask. The nuclear outline was expanded into a doughnut by a fixed length equal to 1/3 of the mean nuclear radius. The cell was classified as positive if the positive pixel density within the doughnut exceeded a pre-defined threshold (CD3:240, CD4:640, CD8:520, and FOXP3:1200). The process was repeated for all antibody channels. Binary masks from the pixel-based segmentation approach were used to analyze T cell populations. Single positive pixels (pixels colored only by one of the antibodies) were counted after excluding double and higher order labeled pixels from individual antibody masks. Double positive pixels (pixels positive for two antibodies) were generated by the intersection of two masks. Triple positive pixels (pixels positive for three or more antibodies) were identified by the overlap of pixels of three masks. Binary masks of cancer cells and fibroblasts were generated from unmixed images of mIF slides. The masks for cancer cells and fibroblasts were obtained by identifying Keratin 8/18 and α-SMA positive cells, respectively. For example, binary masks were obtained with Keratin 8/18 in the foreground (cancer cells mask, white pixels) and the remaining tissue components in the background (black pixels).
Replication	Replicates or independed methods of validation were included
Randomization	Mice were randomized into treatment and control groups. The TMA of patient-matched primary ovarian cancer, synchronous pre-treatment metastasis, and metachronous post-treatment recurrence samples from 42 patients with HGSOC was generated. Each primary, metastatic, and recurrent HGSOC sample was represented by triplicate 1 mm cores that were punched at different locations in the corresponding original FFPE tumor block. The 378 cores were distributed on two slides that were stained at the same time in an automated stainer
Blinding	The investigators were blinded to the study groups and data analyses except in cases where the the analysis was conducted by the Research Core personnel or when the results were determined computationally and the investigator could not influence the outcome

Reporting for specific materials, systems and methods

We require information from authors about some types of materials, experimental systems and methods used in many studies. Here, indicate whether each material, system or method listed is relevant to your study. If you are not sure if a list item applies to your research, read the appropriate section before selecting a response.

Materials & experimental systems

n/a	Involved in the study
☐	☒ Antibodies
☐	☒ Eukaryotic cell lines
☐	☐ Palaeontology and archaeology
☐	☒ Animals and other organisms
☐	☒ Clinical data
☐	☐ Dual use research of concern
☐	☐ Plants

Methods

n/a	Involved in the study
☐	☐ ChIP-seq
☐	☒ Flow cytometry
☐	☐ MRI-based neuroimaging

Antibodies

Antibodies used	IgG (MAB002, R&D Systems)<br>Activin A beta A subunit antibody (mAb3381, R&D Systems)<br>α-SMA antibody (α-am-1, Leica Biosystems)<br>CD8 (clone JCB117, Ventana)<br>CD3 (clone 2GV6, Ventana)<br>CD4 (clone SPO32, Cell Marque)<br>FOXP3 (clone 236A/E7, ThermoFisher Scientific)<br>cytokeratin 8/18 (clones B22.1/B23.1, Ventana)<br>CD8-Alexa Fluor 700 (#344724, BioLegend)<br>CD4-APC/Fire 750 (#300560, BioLegend)<br>CD25-BV510 (#302639, BioLegend)<br>FOXP3-APC (#17-4777-42, eBioscience)<br>The following antibodies (all from Cell Signaling Technologies) were used for Western blot analyses: PD-L1 (#13684), SMAD2 (#5339), pSMAD2 (#3104), SMAD3 (#9523), and pSMAD3 (#9520) and GAPDH (#5174).
Validation	The antibodies were validated by the Pathology Core using tissues known to express specific proteins. Additionally, the TMA had control tissues, including the placenta, ovary, fallopian tube, and tonsil.

Eukaryotic cell lines

Policy information about cell lines and Sex and Gender in Research

Cell line source(s)	Mouse: BR-Luc mouse ovarian cancer cells (genotype: p53-/-, Brca1-/-, myc, Akt) have been dedcribed (Jia D, Nagaoka Y, Katsumata M, and Orsulic S (2018). Inflammation is a key contributor to ovarian cancer cell seeding. Sci Rep 8, 12394.; Taylan E, Zayou F, Murali R, Karlan BY, Pandol SJ, Edderkaoui M, et al. (2020). Dual targeting of GSK3B and HDACs reduces tumor growth and improves survival in an ovarian cancer mouse model. Gynecol Oncol 159, 277.) Syngeneic SO1 mouse ovarian cancer cells have been described in Beach JA, Aspuria PJ, Cheon DJ, Lawrenson K, Agadjanian H, Walsh CS, et al. (2016). Sphingosine kinase 1 is required for TGF-beta mediated fibroblastto- myofibroblast differentiation in ovarian cancer. Oncotarget 7, 4167. Human: OVCAR3 was obtained form ATCC and Kuramochi cells were obtained from the JCRB cell bank.

	781T3 human ovarian cancer fibroblast cells were a gift form Dr. Kate Lawrenson and primary cancer fibroblasts were generated at Cedars-Sinai Medical Center Core for Pathology and Translational Research. Pan T cells were isolated from the peripheral blood of consented healthy donors.
Authentication	The authenticity of the 781T CAFs and Kuramochi ovarian cancer cells was confirmed by Applied Biosystems. The authenticity of OVCAR3 cells was confirmed by Laragen.
Mycoplasma contamination	All cell lines were confirmed to be negative for mycoplasma contamination
Commonly misidentified lines (See ICLAC register)	None of the commonly misidentified cell lines were used in this study

Palaeontology and Archaeology

Specimen provenance	N/A
Specimen deposition	N/A
Dating methods	N/A

☐ Tick this box to confirm that the raw and calibrated dates are available in the paper or in Supplementary Information.

Ethics oversight	N/A

Note that full information on the approval of the study protocol must also be provided in the manuscript.

Animals and other research organisms

Policy information about studies involving animals; ARRIVE guidelines recommended for reporting animal research, and Sex and Gender in Research

Laboratory animals	C67BL/6 and FVBn mice
Wild animals	N/A
Reporting on sex	Only female mice were used to better mimic ovarian cancer in humans, which occurs in females only
Field-collected samples	N/A
Ethics oversight	Cedars-Sinai Medical Center IACUC<br>University of California Los Angeles ARC

Note that full information on the approval of the study protocol must also be provided in the manuscript.

Clinical data

Policy information about clinical studies
All manuscripts should comply with the ICMJE guidelines for publication of clinical research and a completed CONSORT checklist must be included with all submissions.

Clinical trial registration	N/A
Study protocol	N/A
Data collection	N/A
Outcomes	N/A

Dual use research of concern

Policy information about dual use research of concern

Hazards

Could the accidental, deliberate or reckless misuse of agents or technologies generated in the work, or the application of information presented in the manuscript, pose a threat to:

No	Yes	
☒	☐	Public health
☒	☐	National security
☒	☐	Crops and/or livestock
☒	☐	Ecosystems
☒	☐	Any other significant area

Experiments of concern

Does the work involve any of these experiments of concern:

No	Yes	
☒	☐	Demonstrate how to render a vaccine ineffective
☒	☐	Confer resistance to therapeutically useful antibiotics or antiviral agents
☒	☐	Enhance the virulence of a pathogen or render a nonpathogen virulent
☒	☐	Increase transmissibility of a pathogen
☒	☐	Alter the host range of a pathogen
☒	☐	Enable evasion of diagnostic/detection modalities
☒	☐	Enable the weaponization of a biological agent or toxin
☒	☐	Any other potentially harmful combination of experiments and agents

Plants

Seed stocks	N/A
Novel plant genotypes	N/A
Authentication	N/A

ChIP-seq

Data deposition

☐ Confirm that both raw and final processed data have been deposited in a public database such as GEO.

☐ Confirm that you have deposited or provided access to graph files (e.g. BED files) for the called peaks.

Data access links<br>*May remain private before publication.*	N/A
Files in database submission	N/A
Genome browser session<br>(e.g. UCSC)	N/A

Methodology

Replicates	N/A
Sequencing depth	N/A
Antibodies	N/A
Peak calling parameters	N/A
Data quality	N/A
Software	N/A

Flow Cytometry

Plots

Confirm that:

☒ The axis labels state the marker and fluorochrome used (e.g. CD4-FITC).

☒ The axis scales are clearly visible. Include numbers along axes only for bottom left plot of group (a 'group' is an analysis of identical markers).

☒ All plots are contour plots with outliers or pseudocolor plots.

☒ A numerical value for number of cells or percentage (with statistics) is provided.

Methodology

Sample preparation	The tumor tissue was manually minced with a sterile scalpel and enzymatically digested by rotating at 37°C for 30 minutes with 1 mg/ml Collagenase/Dispase (Roche). The cells were isolated by filtering the tissue through an 80 µm filter, centrifugation at 400 g for 8 minutes, lysing red blood cells (8 g NH4Cl, 1 g KHC03 in 1L H2O, pH adjusted to 7.2), and another centrifugation at 400 g for 5 minutes. The cell pellet was washed with a wash buffer twice and filtered through an 80 µm filter, followed by additional filtration through a 40 µm filter. The cells were seeded in a 10 cm dish and cultured at 37°C in 5% CO2 until confluent. Flow cytometry analysis was conducted to confirm that the cells were CD45(-) CD31(-) CD326 (-) (PE/Cy7 anti-human CD31, BioLegend #303118; Alexa Fluor 647 anti-human CD326, Biolegend #324212; Brilliant Violet 421™ anti-human CD45, BioLegend #304032). For analysis of cells by flow cytometry, all cells were incubated with Live/Dead Fixable Blue stain (#L23105, Thermo Fisher Scientific) for 15 minutes at 4°C. Surface antibody staining (anti-human CD4, CD8, CD25) was performed for 30 minutes at 4°C, followed by fixation/permeabilization for 30 minutes at 4°C (FOXP3 / Transcription Factor Staining Buffer Set Kit, #00-5523-00, eBioscience). Cells were then stained for intracellular factor FOXP3 for 30 minutes at room temperature. The following antibodies were used: CD8-Alexa Fluor 700 (#344724, BioLegend), CD4-APC/Fire 750 (#300560, BioLegend), CD25-BV510 (#302639, BioLegend), and FOXP3-APC (#17-4777-42, eBioscience). All samples were acquired with an LSRII flow cytometer and analyzed with FlowJo software (version 10.4.0) and a multistep gating strategy to identify immune cells.
Instrument	LSRII flow cytometer
Software	FlowJo software (version 10.4.0)
Cell population abundance	Only cells with >90% purity were used
Gating strategy	Multistep gating strategy was provided by the Flow Cytometry Core personnel experienced in gating protocols with different antibodies

☐ Tick this box to confirm that a figure exemplifying the gating strategy is provided in the Supplementary Information.

Magnetic resonance imaging

Experimental design

Design type	N/A
Design specifications	N/A
Behavioral performance measures	N/A

Acquisition

Imaging type(s)	N/A
Field strength	N/A
Sequence & imaging parameters	N/A
Area of acquisition	N/A

Diffusion MRI ☐ Used ☒ Not used

Preprocessing

Preprocessing software	N/A
Normalization	N/A

Normalization template | N/A

Noise and artifact removal | N/A

Volume censoring | N/A

Statistical modeling & inference

Model type and settings | N/A

Effect(s) tested | N/A

Specify type of analysis: ☐ Whole brain ☐ ROI-based ☐ Both

Statistic type for inference (See Eklund et al. 2016) | N/A

Correction | N/A

Models & analysis

n/a	Involved in the study
☒	☐ Functional and/or effective connectivity
☒	☐ Graph analysis
☒	☐ Multivariate modeling or predictive analysis
