# Supplementary material for: INHBA(+) cancer-associated fibroblasts generate an immunosuppressive tumor microenvironment in ovarian cancer
Source: NPJ Precis Oncol. 2024 Feb 15;8:35. doi: 10.1038/s41698-024-00523-y (PMC10869703; doi:10.1038/s41698-024-00523-y)

SUPPLEMENTARY FIGURE LEGEND

Supplementary Figure 1. Distribution of inhibin genes in cancer and corresponding normal tissues. a Box plots of expression levels in cancers (red) and normal tissues (green) for genes in the inhibin family. Of note, only INHBA is significantly (p<0.001) overexpressed in cancers compared to normal tissues (red and green arrows point to dotted lines that indicate average expression levels across different cancer types and normal tissues. **b** INHBA encodes inhibin βA chain, which is present as a homodimer in Activin A, a heterodimer with inhibin βB in Activin AB, and a heterodimer with inhibin α in Inhibin A. Since genes encoding the heterodimer components (INHBB and INHA) are not overexpressed in cancer compared to normal tissues, we can conclude that only Activin A protein (encoded by a homodimer of INHBA) is overexpressed in cancer.

Supplementary Figure 2. INHBA, but not ACTA2, mRNA is associated with poor survival in ovarian cancer. Ovarian cancer overall survival (n=1868 patients) and disease-free survival (n=1516 patients) based on expression levels of **a** INHBA and **b** ACTA2 (encodes α-SMA). The Kaplan-Meier plots and associated data were obtained from the http://csibio.nus.edu.sg/CSIOVDB/CSIOVDB.html database (Tan TZ, Miow QH, Huang RY, et al., Functional genomics identifies five distinct molecular subtypes with clinical relevance and pathways for growth control in epithelial ovarian cancer. *EMBO Mol Med.* 2013, 7:983-98).

Supplementary Figure 3. INHBA is expressed in cancer-associated fibroblasts. a A human HGSOC tissue microarray stained with α-SMA antibody (α-am-1, Leica Biosystems) by immunohistochemistry (IHC) and human INHBA probe by in situ hybridization (ISH). Squares in the cores in the top panel are magnified in the bottom panel. **b** ISH with a mouse INHBA probe in the BR-luc syngeneic mouse ovarian cancer. **c** INHBA expression levels in laser-capture-microdissected epithelial cells and fibroblasts from normal ovary and primary high-grade serous ovarian carcinoma (GSE40595 dataset; the graph was generated using R2: Genomics Analysis Visualization Platform).

Supplementary Figure 4. INHBA is expressed in a subset of cancer-associated fibroblasts. T-SNE plots of INHBA and ACTA2 mRNA expression in ovarian cancer stroma and mixed cancer stroma. The images were generated using SCope software and public single-cell RNA sequencing data deposited into the ArrayExpress database at EMBL-EBI under accession numbers E-MTAB-8107, E-MTAB-6149, and E-MTAB-6653.

Supplementary Figure 5. INHBA is expressed in a subset of cancer-associated fibroblasts in the tumor microenvironment (TME) of ovarian cancer. **a** INHBA and **b** ACTA2 RNA expression in the UMAP of TME of the MSK SPECTRUM cohort HGSOC at single-cell resolution. **c** Cells are colored based on cell type. **d** Cells are colored based on the sample site. Fibroblasts in the ascites show negligible expression levels of INHBA. The images were generated using SCope software and public single-cell RNA sequencing data. The data were downloaded and visualized via Synapse (accession number syn25569736: https://www.synapse.org/msk_spectrum).

Supplementary Figure 6. Representative INHBA ISH and α-SMA IHC staining of the tissue microarray (TMA) of primary, metastatic, and recurrent high-grade serous ovarian tumors. Each matched tumor is represented by three cores cut at different places in the tumor block.

Supplementary Figure 7. Multiplex immunofluorescence (mIF) data analysis in the ovarian cancer TMA. **a** Strategy for computational analysis of mIF data. **b** An example depicting CD3+CD4+ T cells, CD3+CD8+ T cells as well as CD3+CD4+FOXP3+ Tregs.

SUPPLEMENTARY FIGURE LEGEND (continued)

Supplementary Figure 8. INHBA expression is correlated with the signature of regulatory T cells (Tregs). The data are derived from 130 high-grade serous ovarian cancer metastases to the omentum (GSE138866). **a** Correlation between the proportion of immunocytes and fibroblasts (left) or immunocytes and epithelial cancer cells (right). **b** Correlation between INHBA expression and proportion of fibroblasts, epithelial cancer cells, and immunocytes. **c** Correlation between INHBA expression and immune cell metagenes for CD4, CD8, and Treg cells. Statistically significant p values are outlined in red. The graphs were generated using R2: Genomics analysis and visualization platform. https://r2.amc.nl.

Supplementary Figure 9. Intraperitoneal injection of INHBA shRNA attenuates *in vivo* tumor growth. **a** Intravital bioluminescence signal quantification of tumor burden in the BR-luc immunocompetent mouse ovarian cancer model. The syngeneic mouse ovarian cancer cell line BR-luc ($1x10^6$) was implanted into FVB mice. Six days later, the mice were injected i.p with control shRNA or INHBA shRNA (100 µg shRNA per mouse) 2 times per week for 30 days. Tumor burden in both groups (n=5 per group) was quantified by IVIS imaging 30 days after the first injection of shRNA. **b** Detection of INHBA (+) cells in mouse tumors by in situ hybridization.

Supplementary Figure 10. INHBA and CD274 (PD-L1) are co-expressed in myCAFs. Expression levels of INHBA and CD274 in different cell types in **a** DUOS-000002 and **b** PRJEB35405 single-cell RNA sequencing datasets.

Supplementary Figure 11. Anti-PD-L1 therapy (atezolizumab) non-responders express higher levels of INHBA compared to responders (EGAS00001002556 dataset of metastatic urothelial carcinoma patients, n=368).

Supplementary Figure 12. INHBA levels determined by qRT-PCR in samples that were grown in culture in parallel with samples used for Western blots shown in Figs. 5a and 5b in the main text.

Supplementary Figure 13. Identification of Smad binding elements "caga" (highlighted) in the PD-L1 promoter.

Supplementary Figure 14. Gating strategy for Fig.4c in the main text.

Supplementary Figure 15. Gating strategy for Fig. 5d in the main text.

Supplementary Figure 16. Uncropped Western blots for Figs. 5a and b in the main text.

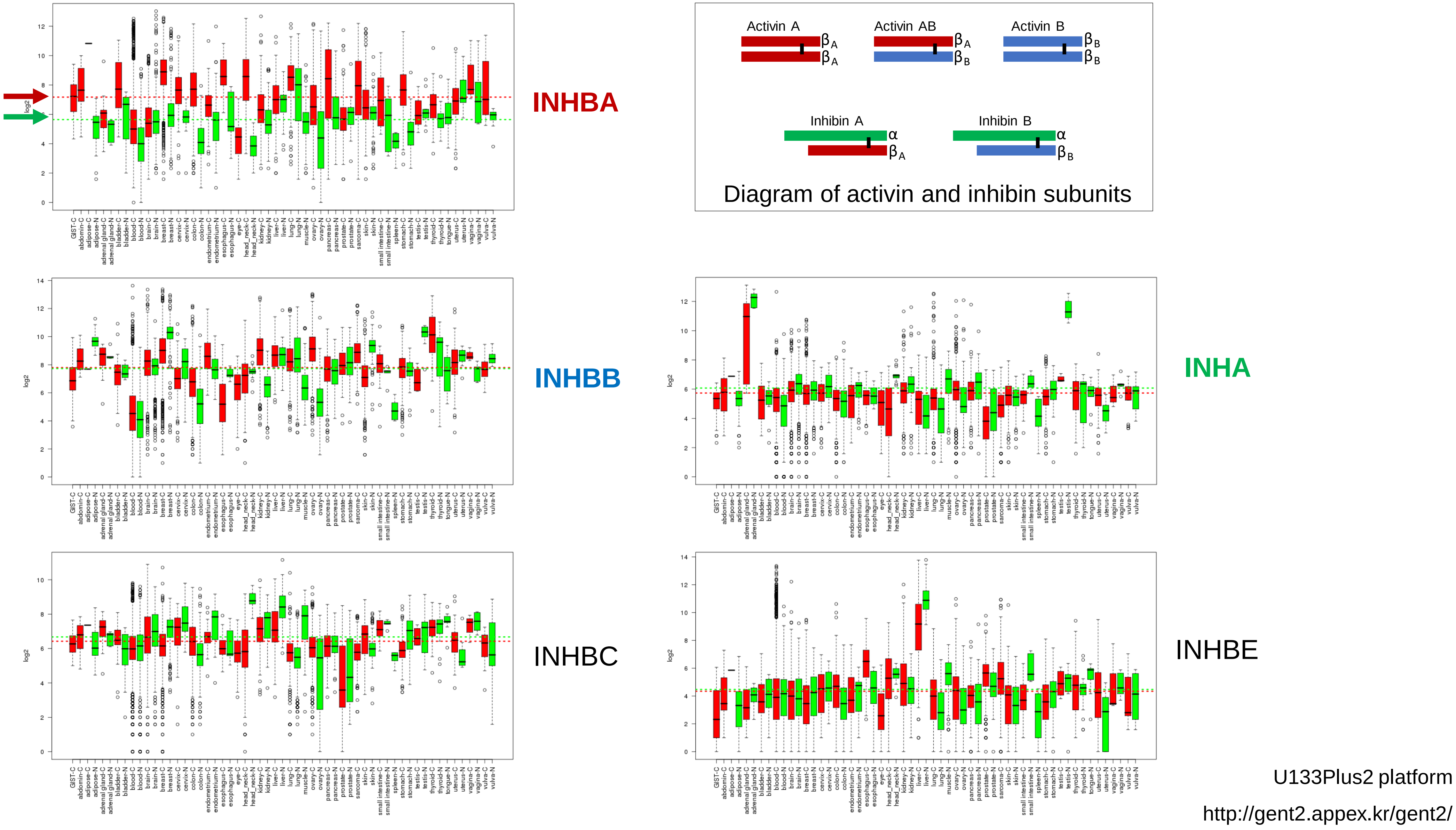

Supplementary Figure 1. Distribution of activins and inhibins in normal tissues and cancer

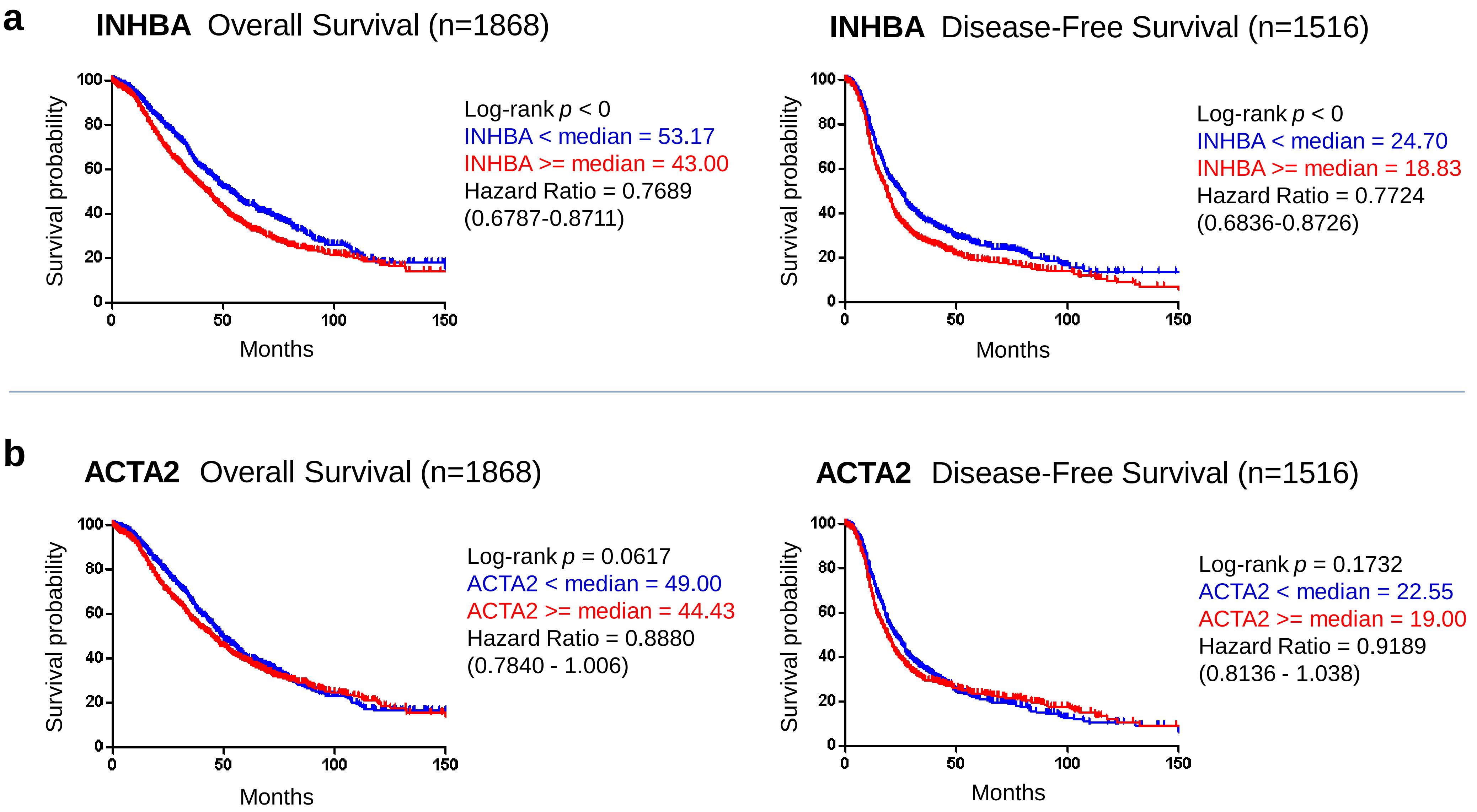

Genes encoding other subunits of activins and inhibins, INHBB, INHBC, INHBE, and INHA, are not associated with survival in ovarian cancer (http://csibio.nus.edu.sg/CSIOVDB/CSIOVDB.html, data not shown).

Supplementary Figure 2. INHBA, but not ACTA2, mRNA is associated with poor survival in ovarian cancer

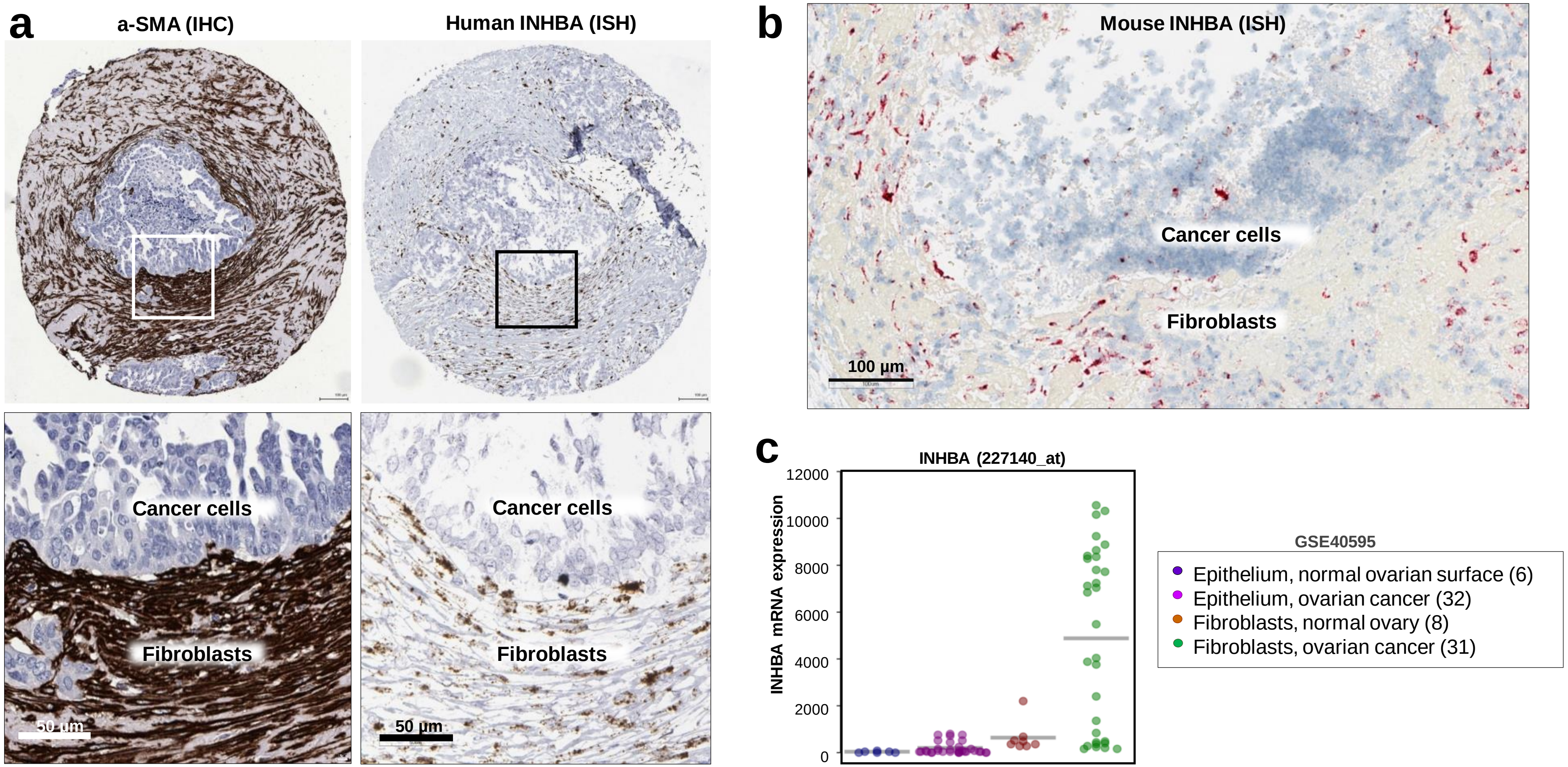

Supplementary Figure 3. INHBA is expressed in cancer-associated fibroblasts

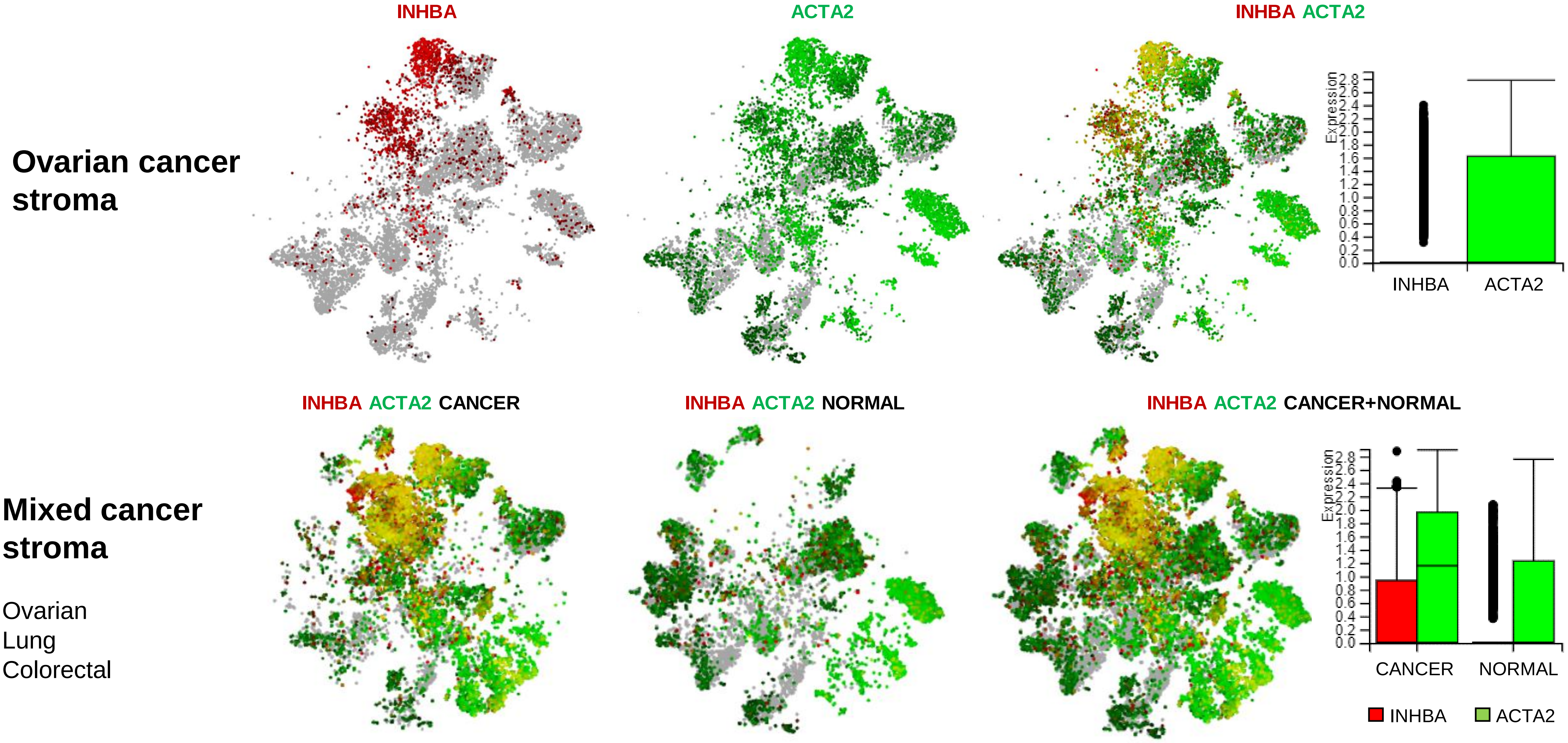

Supplementary Figure 4. INHBA is expressed in a subset of cancer-associated fibroblasts

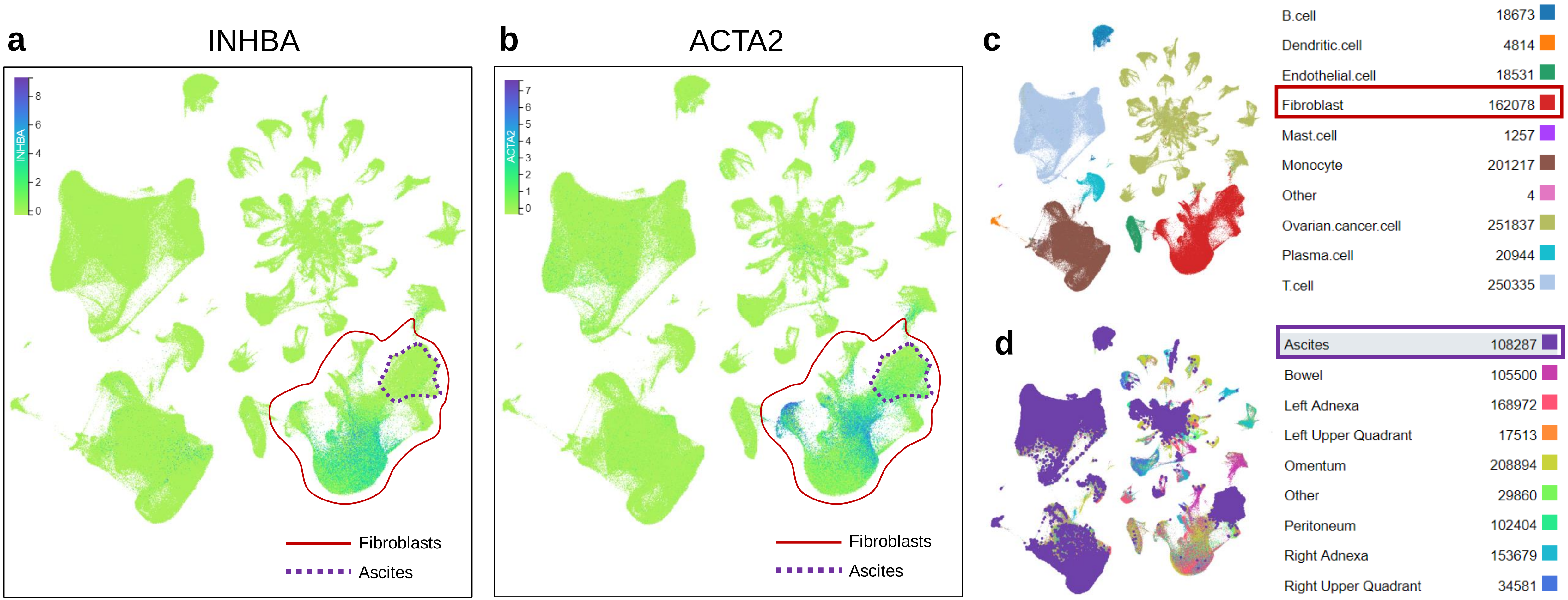

Supplementary Figure 5. INHBA is expressed in a subset of cancer-associated fibroblasts in the tumor microenvironment (TME) of ovarian cancer

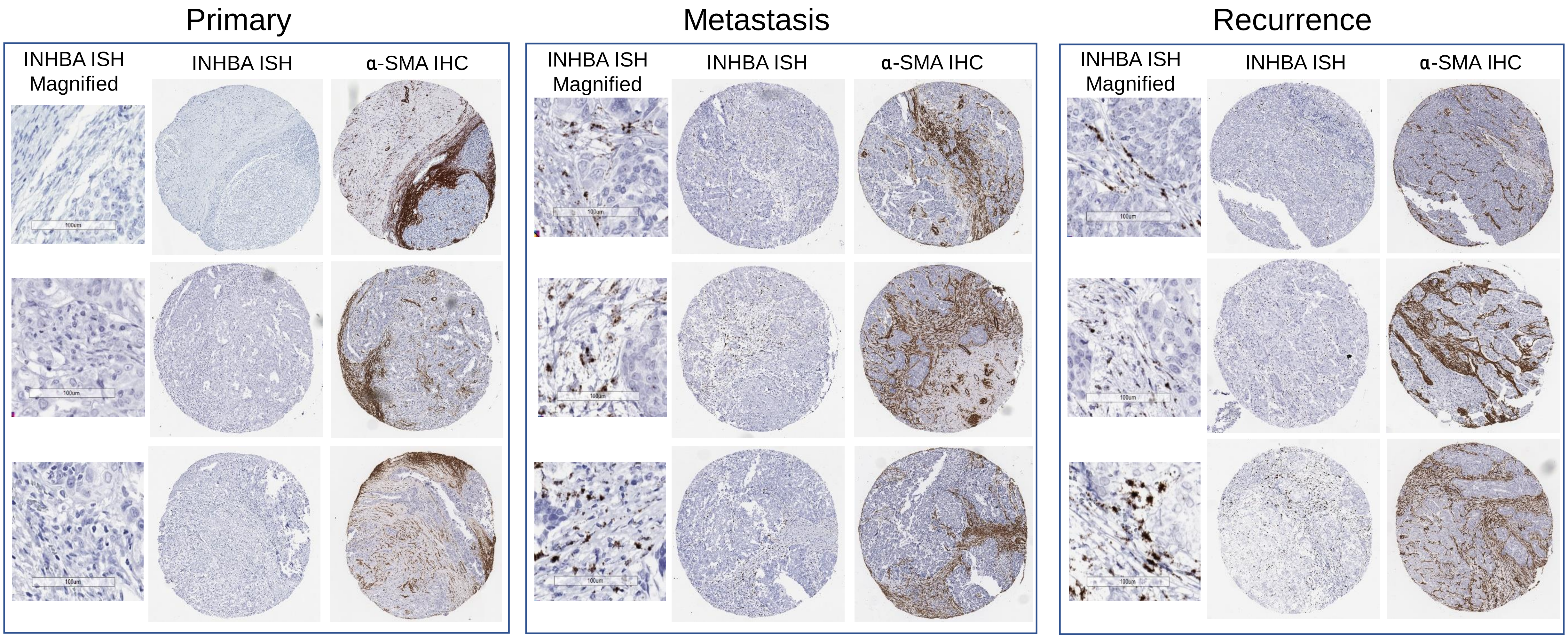

Supplementary Figure 6. Representative INHBA ISH and α-SMA IHC staining in primary, metastatic, and recurrent high-grade serous ovarian tumors

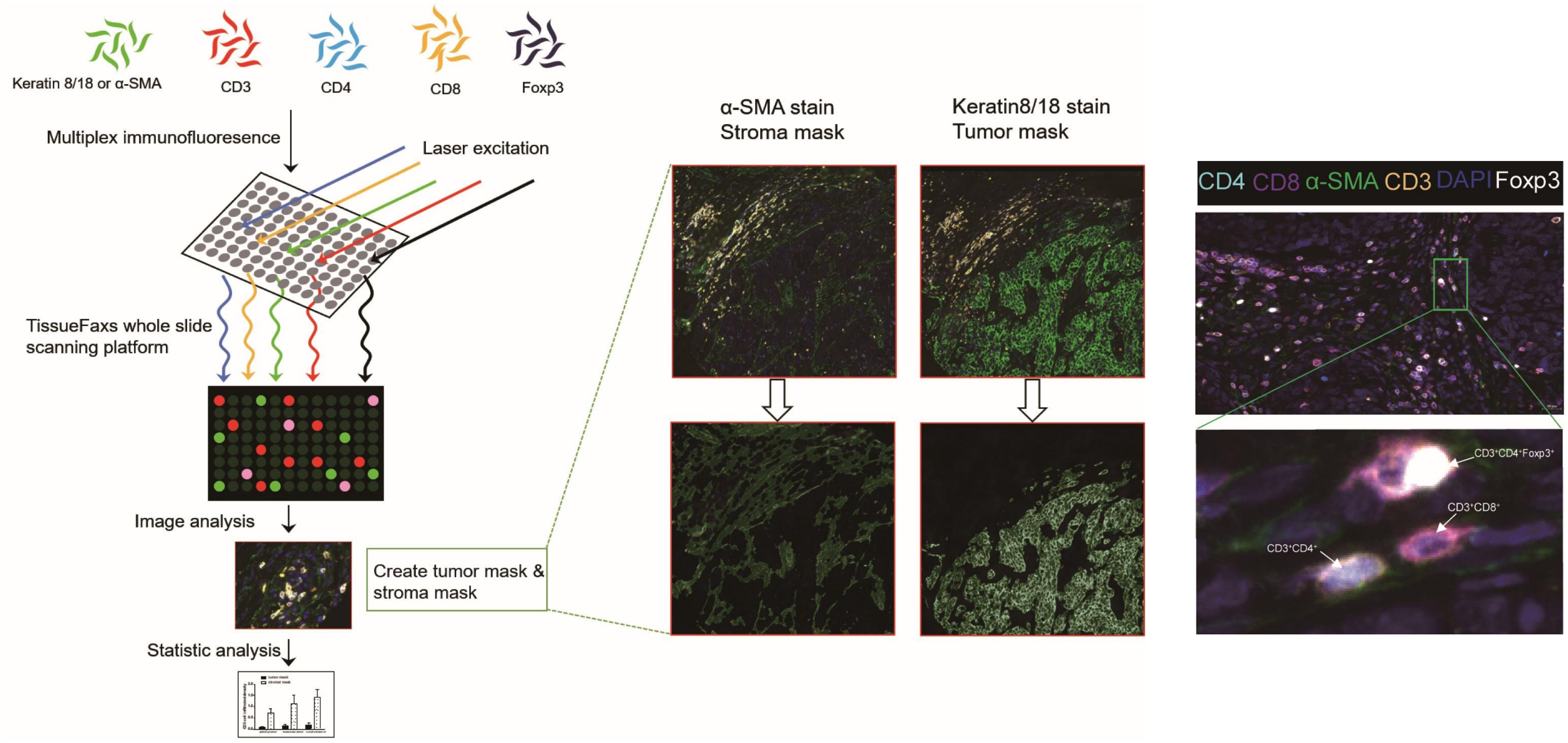

Supplementary Figure 7. Multiplex immunofluorescence (mIF) data analysis in the ovarian cancer TMA

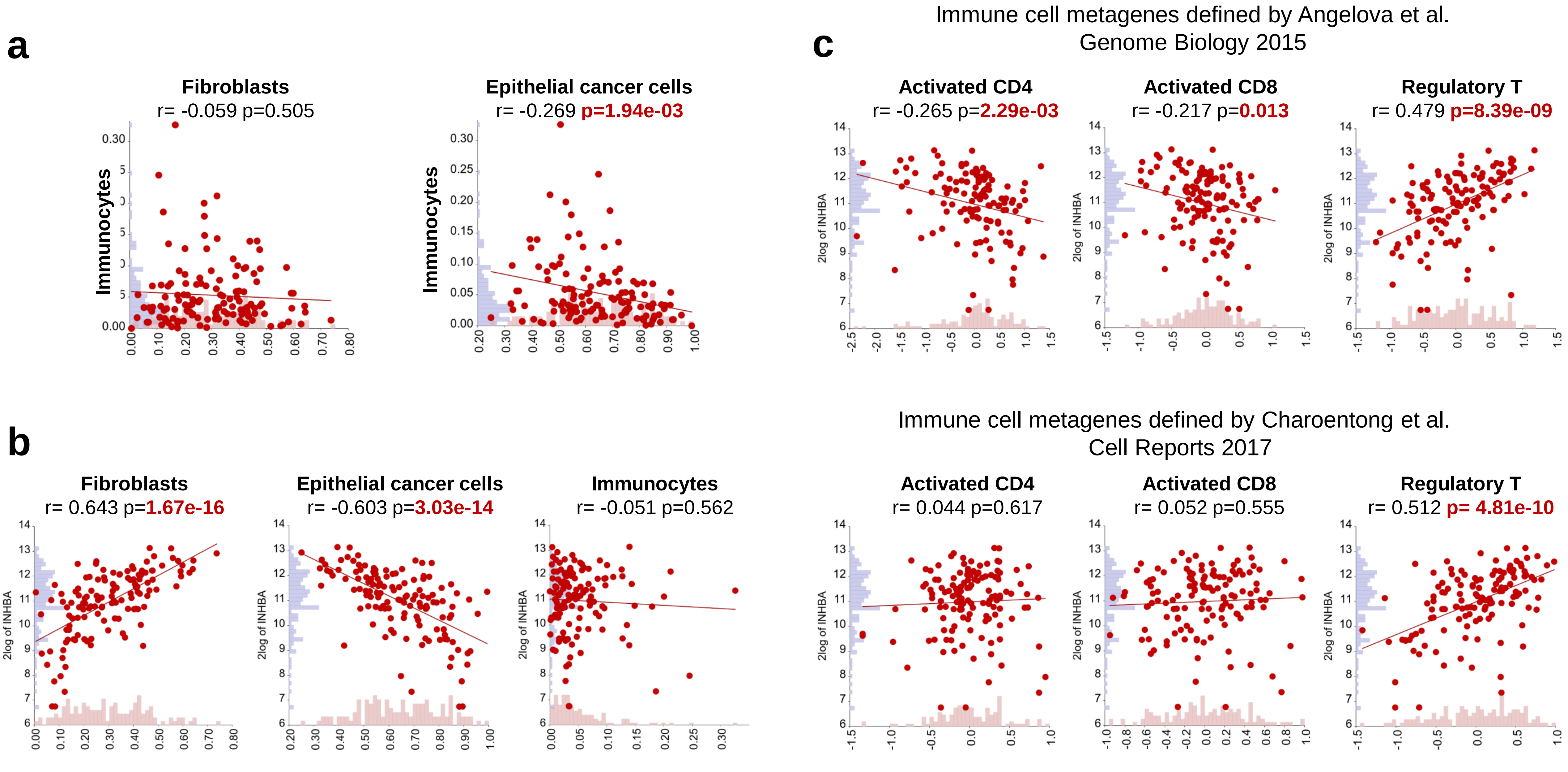

Supplementary Figure 8. INHBA expression is correlated with the signature of regulatory T cells

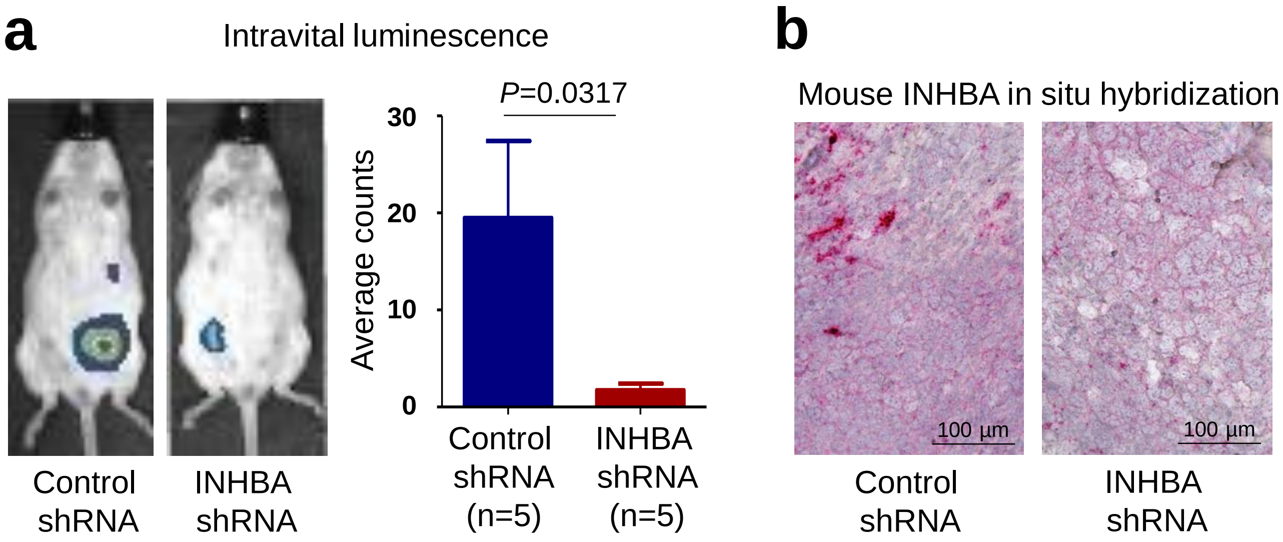

Supplementary Figure 9. Intraperitoneal injection of INHBA shRNA attenuates in vivo tumor growth

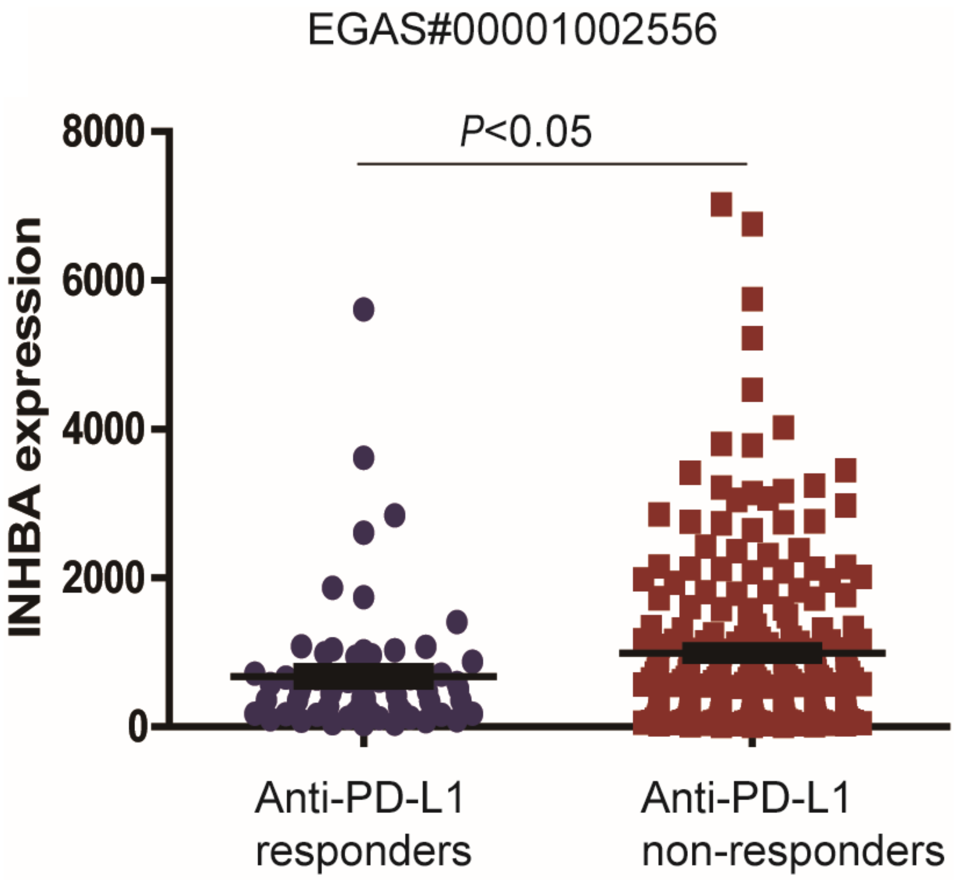

Supplementary Figure 10. Anti-PD-L1 non-responders express higher levels of INHBA

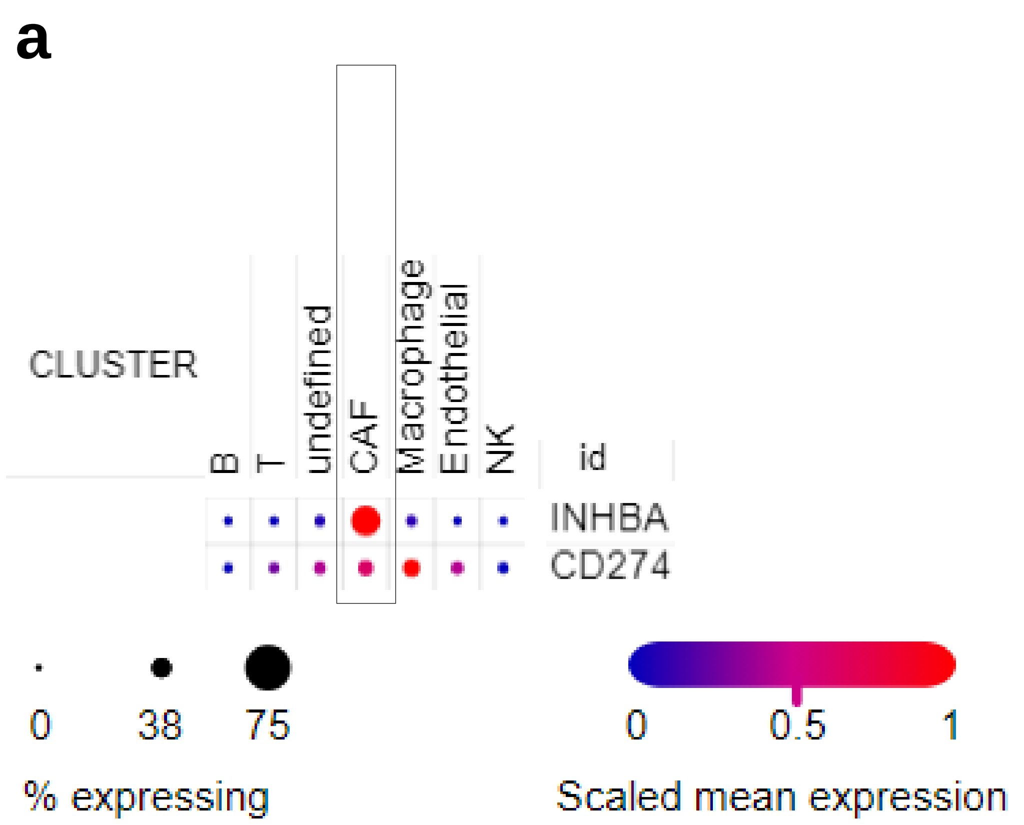

DUOS-000002 dataset: Dissecting the multicellular ecosystem of metastatic melanoma by single-cell RNA-seq.

Tirosh et al. Science 2018

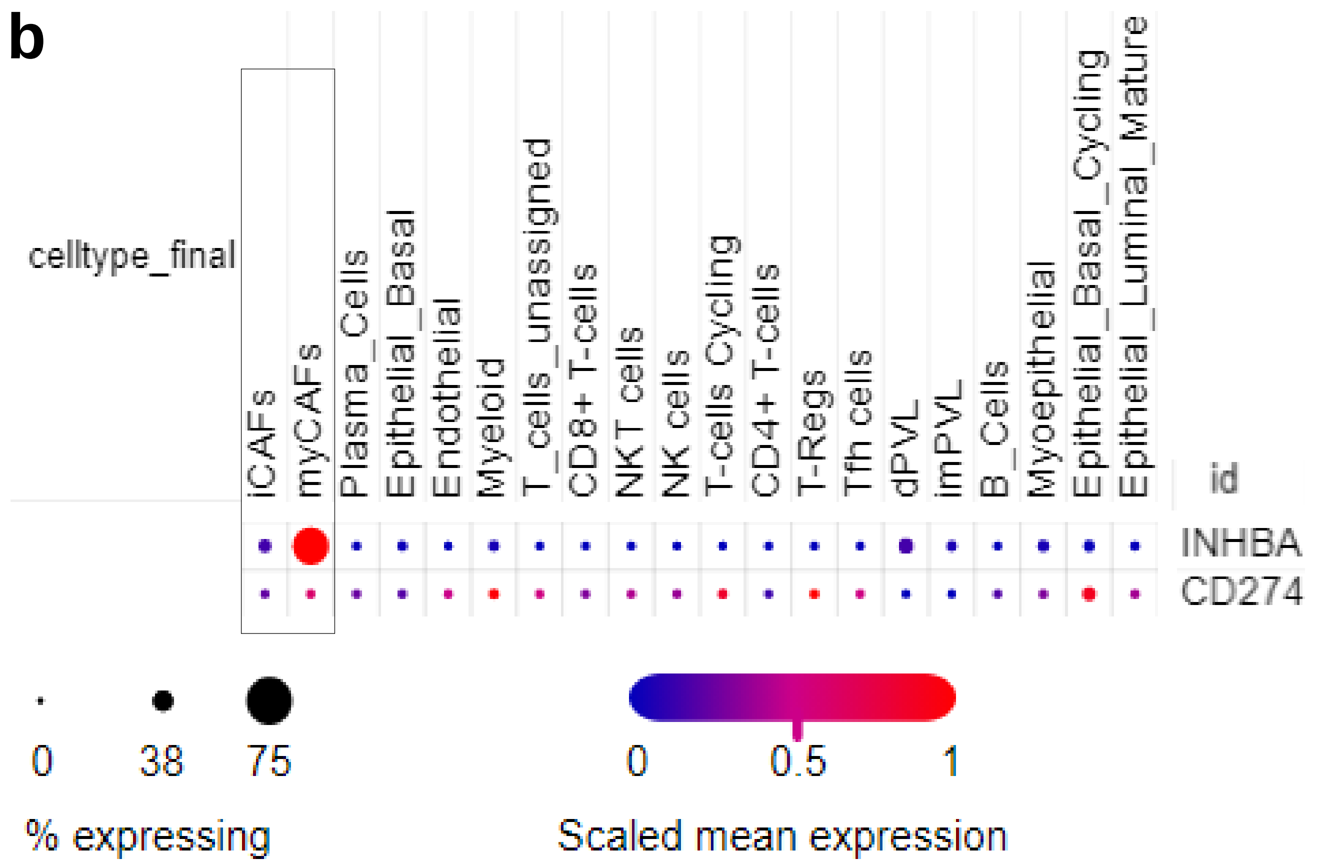

PRJEB35405 dataset: Stromal cell diversity associated with immune evasion in human triple-negative breast cancer.

Wu et al. EMBO J 2020

Supplementary Figure 11. INHBA and CD274 (PD-L1) are co-expressed in myCAFs

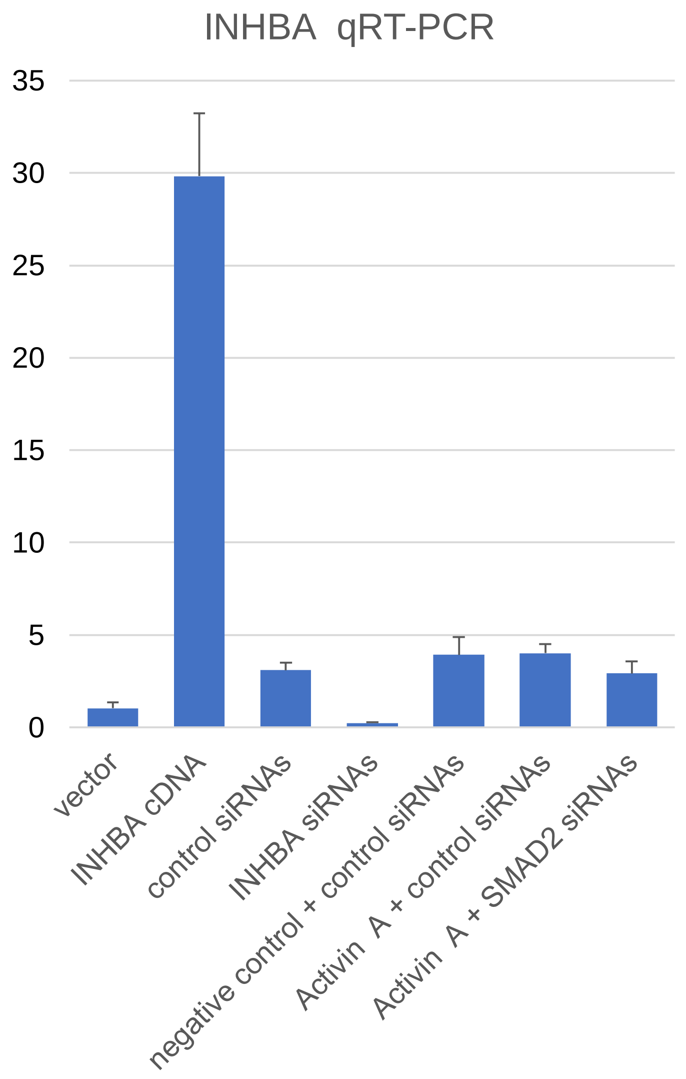

Supplementary Figure 12. INHBA levels determined by qRT-PCR in samples that were grown in culture in parallel with samples used for Western blots shown in Figs. 5a and 5b

```
tttctctttttctaaacacagcctgtttttcaatctccgggtagttgatc
aattgtatgggaaaatgaatggctgaagggtagaaacaggtgggaaagat
gaacaaaaacacgaatcctcacattactaatacgcaaatcactgagcagc
aagctgagcaaataccctcaattcccatcagcaactttagagaaaggcaa
attccgtttgcctcattgatcatttaggtagaccctgaacactgctttca
taaaacaaaaacaaaatacccatccccagtttaaaaaattattcatagat
catccaggccatctaggaggatatgatttaatcctggctacttggtaaat
tatttgcccaagttaactcagctagttagtggtagatggctctgaagcca
gttgtttttttttgttttgtttttgcagacctcaagagtcatgatgaac
tagcagatcataaagtttatgccctgggtcttgaccatttttagaaaaat
aaaacattaaatgaaaatatcagagggcattgcagatagtagatctaagt
atttttcatgaaacttgttgtacatgtgtgtgtcatacacagactatat
atatgcagtacctgtaaactgtattgccacataatgtctatattttccta
gaggtcacagtcaccaaagttgggaagtcacccaacttcgggaactttgg
gaagtcacccaaacttacagtcaccaaaattgctctattctactatgtga
cctcaaaagtgatttgaaagaaggaacatctgagctgggcccaaacccta
ttgcaattttattggggccaaagagaactccatgctcctgccaaatcaag
gcagtgtcagcctcaataatttcccagataaaaataaaaatctgtgatac
aatcagaatgtgaaaattcttattttggaagcaaatgtcataaccaatgc
aagggctatctcaatattcattcattatgcagtattttgaactgcagttg
aaatgaataagaaggaaaggcaaacaacgaagagtccaatttctcaattt
agaaaaagagaaaaaaaaagaaaagggagcacacaggcacggtggctcaag
cctgtaatatcagcactttggcggatcacttgaggtcaaggagttcgaga
aaagagagcacctagaagttcagcgcgggataatacttaagtaaattatg
acaccatcgtctgtcatcttgggcccattcactaacccaaagctttcaaa
agggctttcttaaccctcacctagaataggcttccgcagccttaatcctt
agggtggcagaatatcagggaccctgagcattcttaaaagatgtagctcg
ggatgggaagttcttttaatgacaaagcaaatgaagtttcattatgtcga
ggaactttgaggaagtcacagaatccacgatttaaaaatatatttcctat
tatacacccatacacacacacacacacctactttctagaataaaaaccaa
agccatatgggtctgctgctgactttttatatgttgtagagttatatcaa
gttatgtcaagatgttcagtcaccttgaagaggcttttatcagaaagggg
gacgcctttctgataaaggttaaggggtaaccttaagctcttacccctct
gaaggtaaaatcaaggtgcgttcagatgttggcttgttgtaaatttcttt
ttttattaataacatactaaatgtggatttgctttaatcttcgaaactct
tcccggtgaaaatctcatttacaagaaaactggactgacatgtttcactt
tctgtttcatttctatacacagctttattcctaggacaccaacactagat
acctaaactgaaagcttccgccgatttcaccgaaggtcaggaaagtccaa
cgcccggcaaactggatttgctgccttgggcagaggtgggcgggaccccg
cctccgggcctggcgcaacgctgagcagctggcgcgtcccgcgcggcccc
```

Supplementary Figure 13. Identification of Smad binding elements “caga” (highlighted) in the PD-L1 promoter

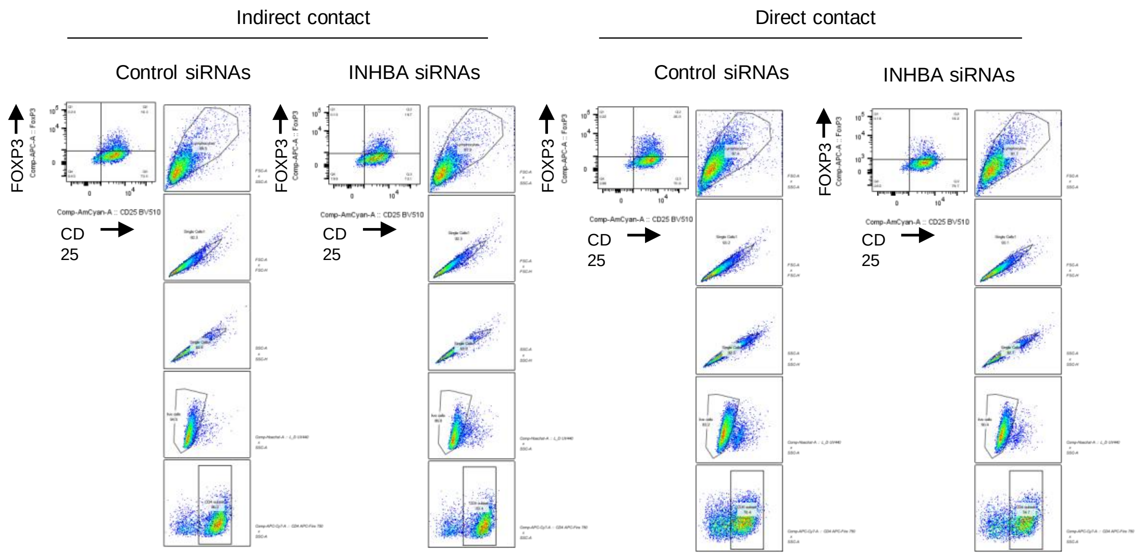

Supplementary Figure 14. Gating strategy for Fig. 4c

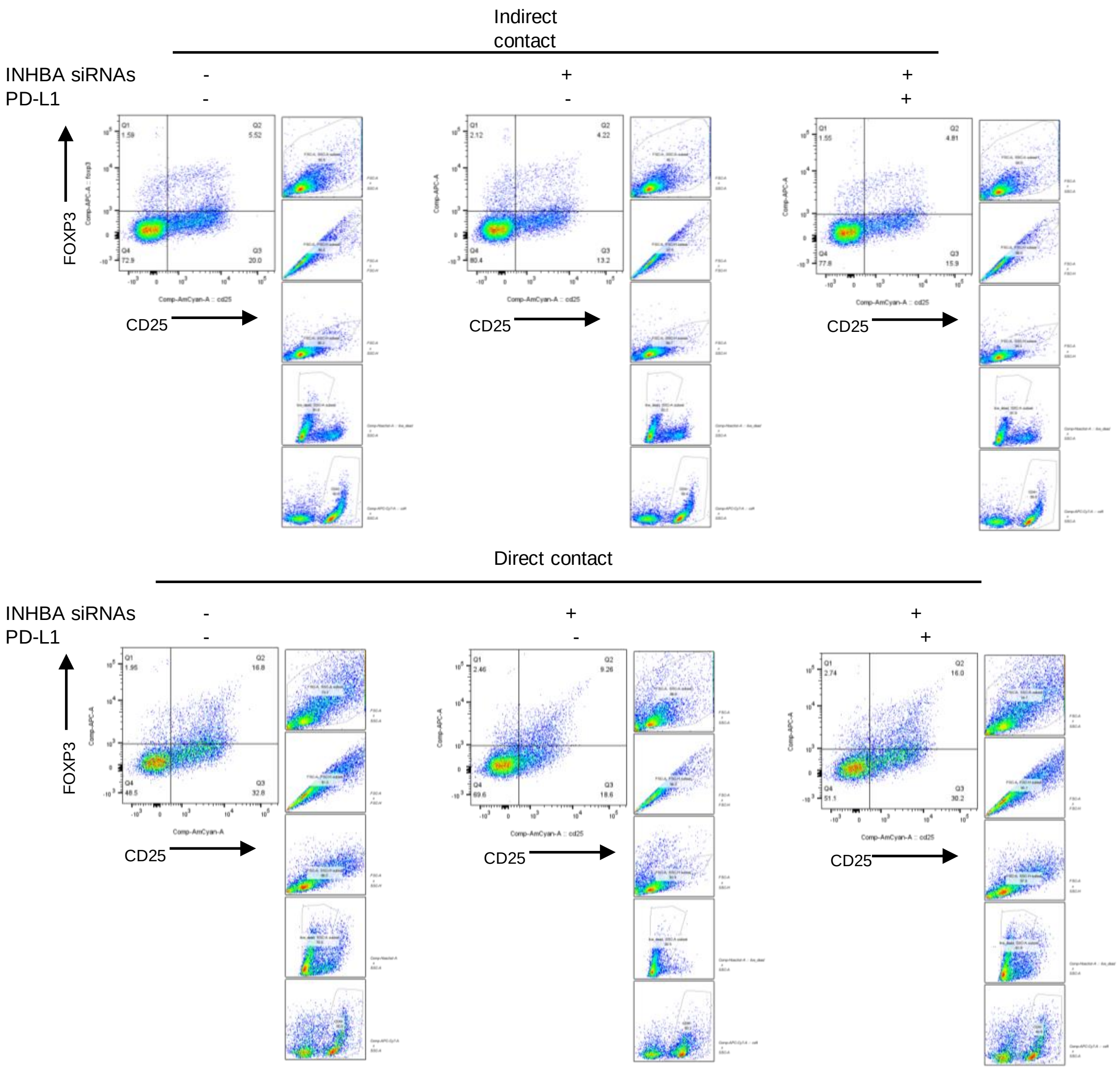

Supplementary Figure 15. Gating strategy for Fig. 5d

Supplementary Figure 16. Uncropped Western Blots for Fig. 5

(next 8 pages)

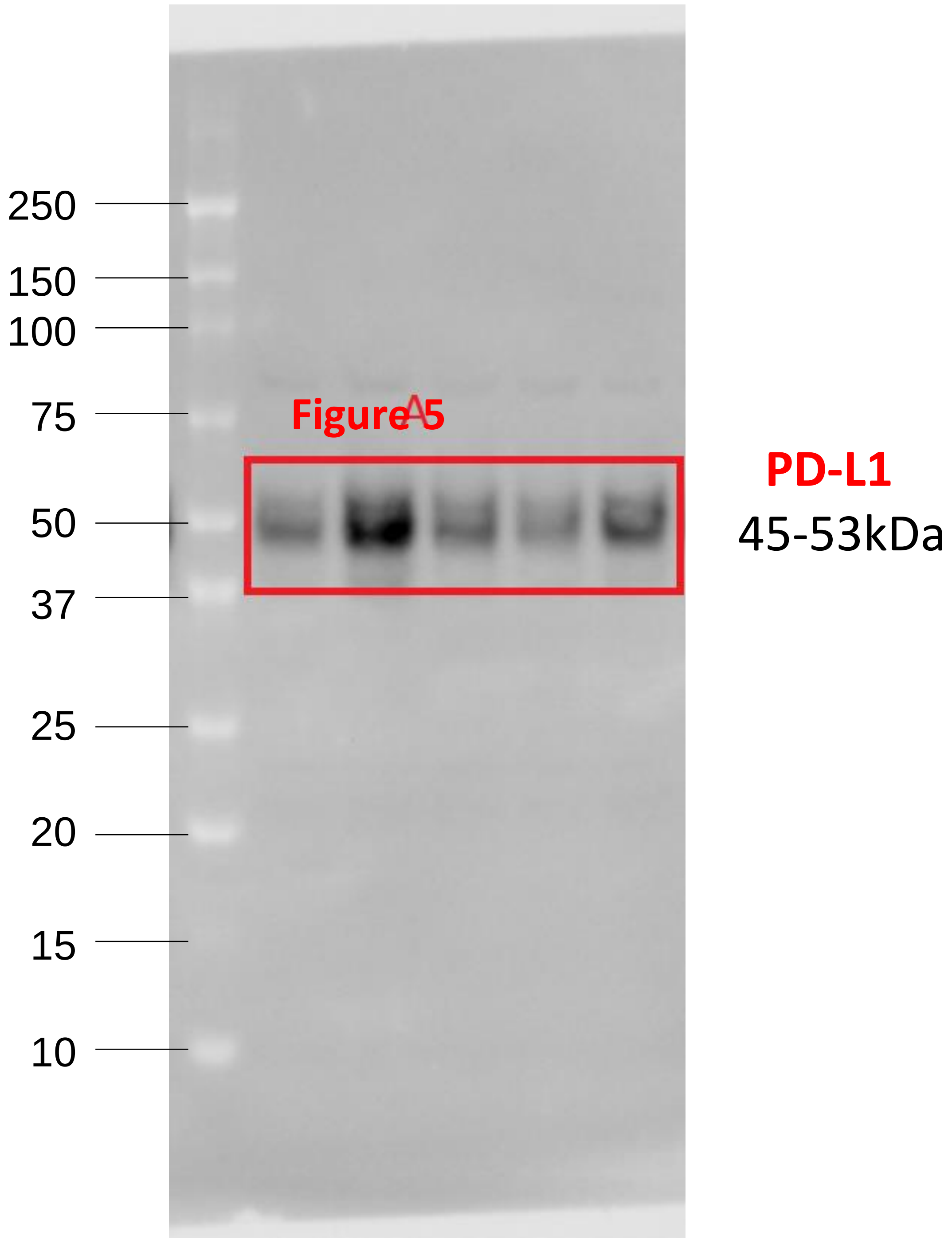
250
150
100
75
50
37
25
20
15
10
Figure 5
PD-L1
45-53kDa

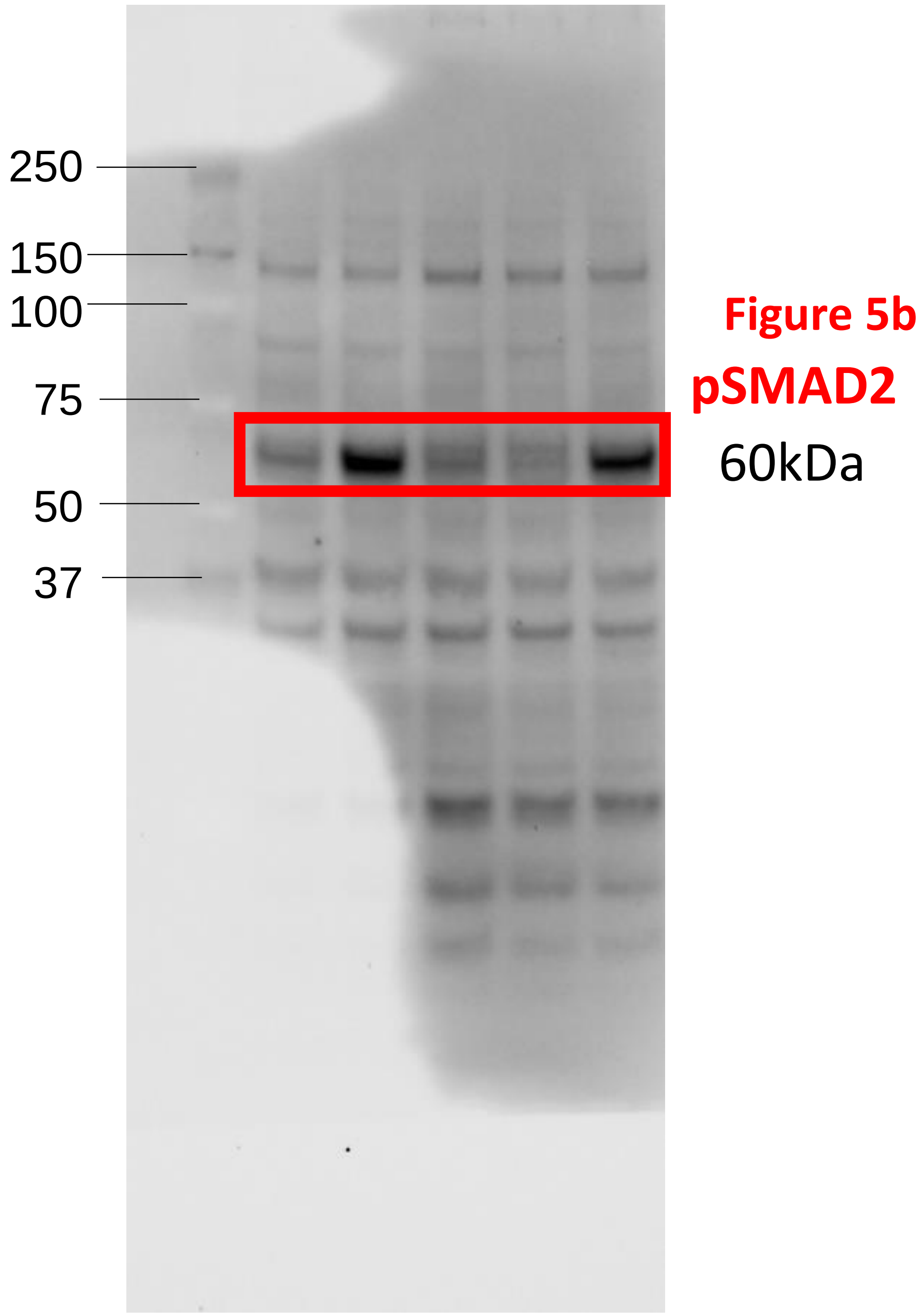
250
150
100
75
50
37
Figure 5b
pSMAD2
60kDa

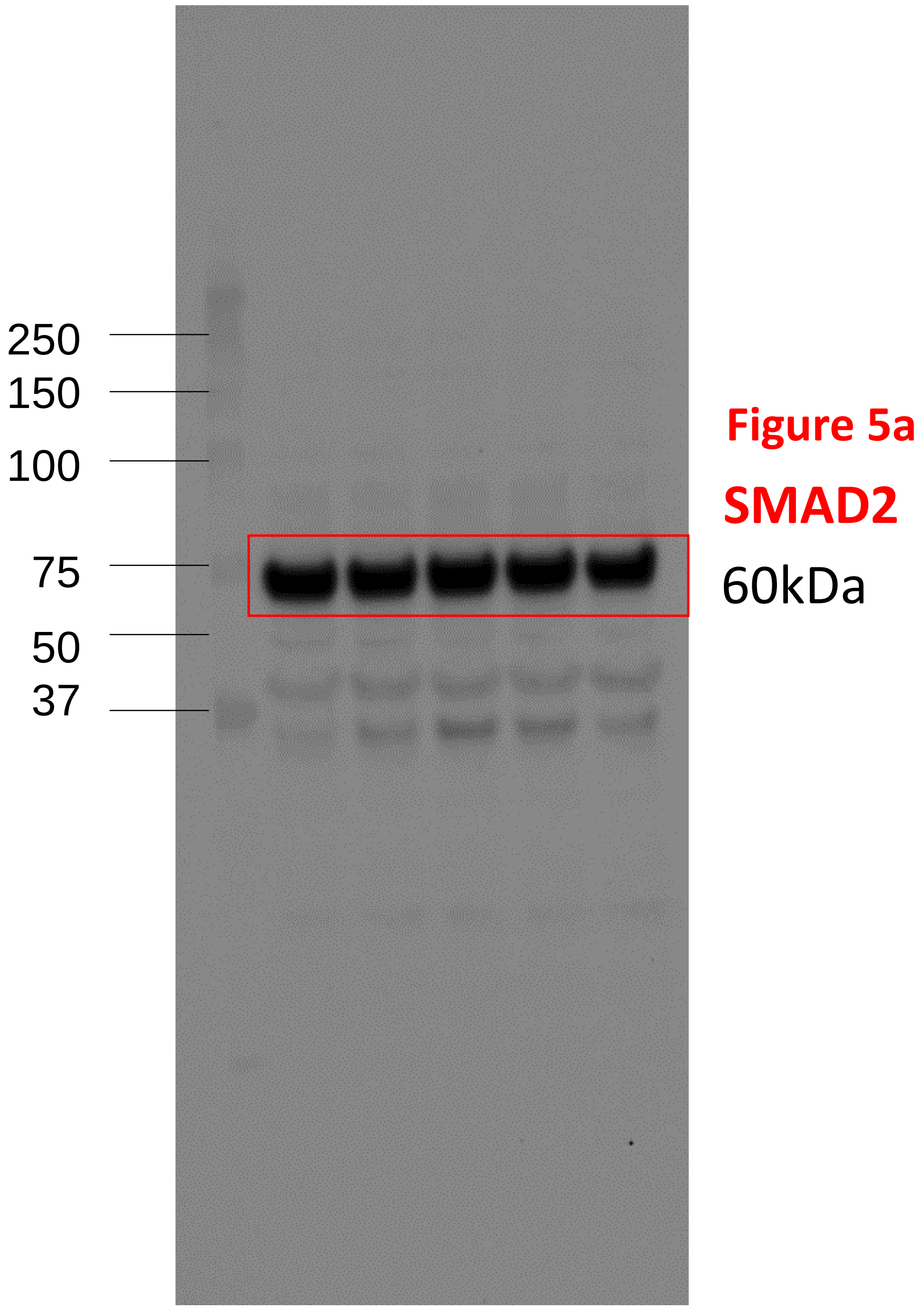

250
150
100
75
50
37
Figure 5a
SMAD2
60kDa

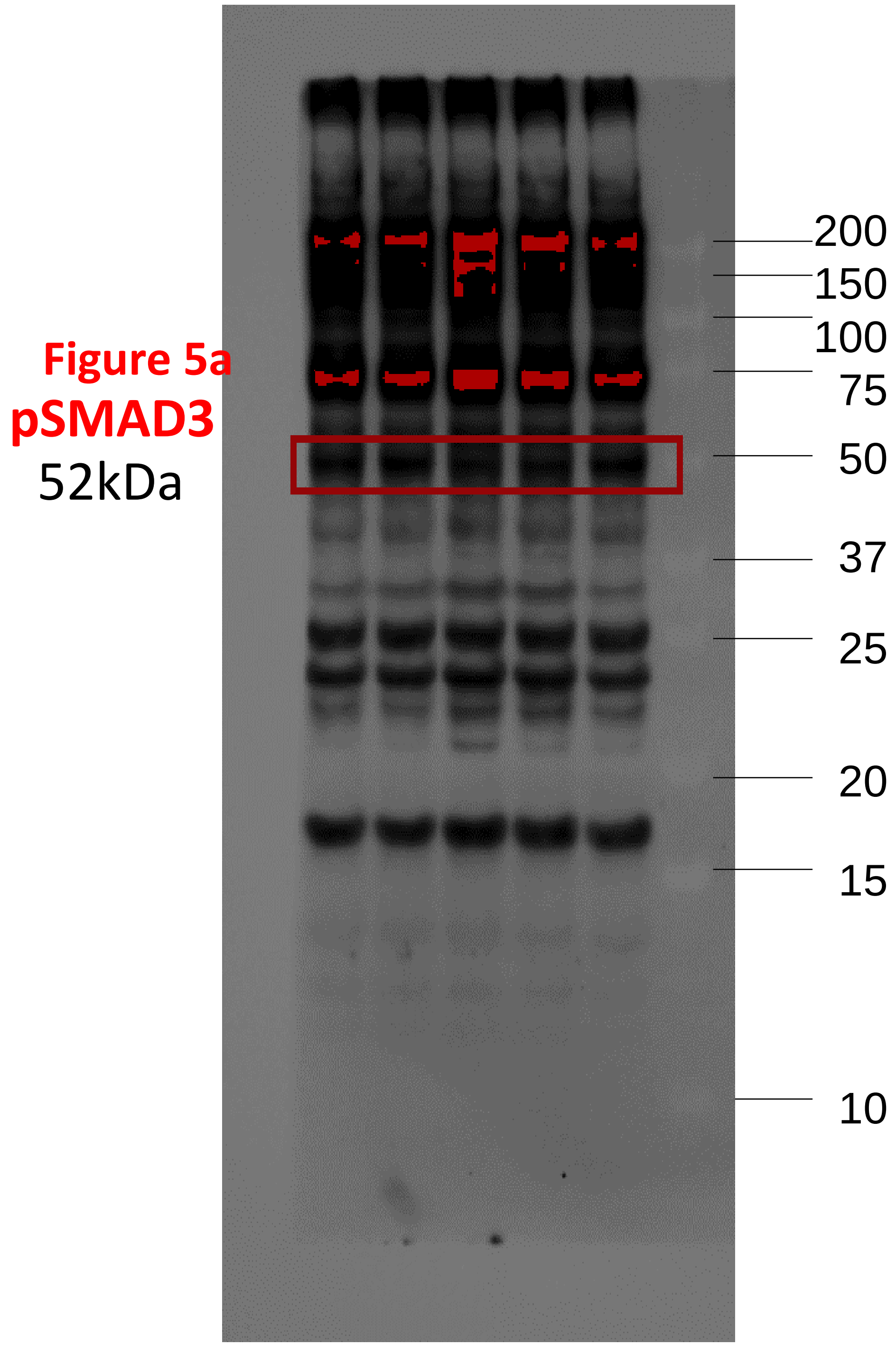
Figure 5a
pSMAD3
52kDa
200
150
100
75
50
37
25
20
15
10

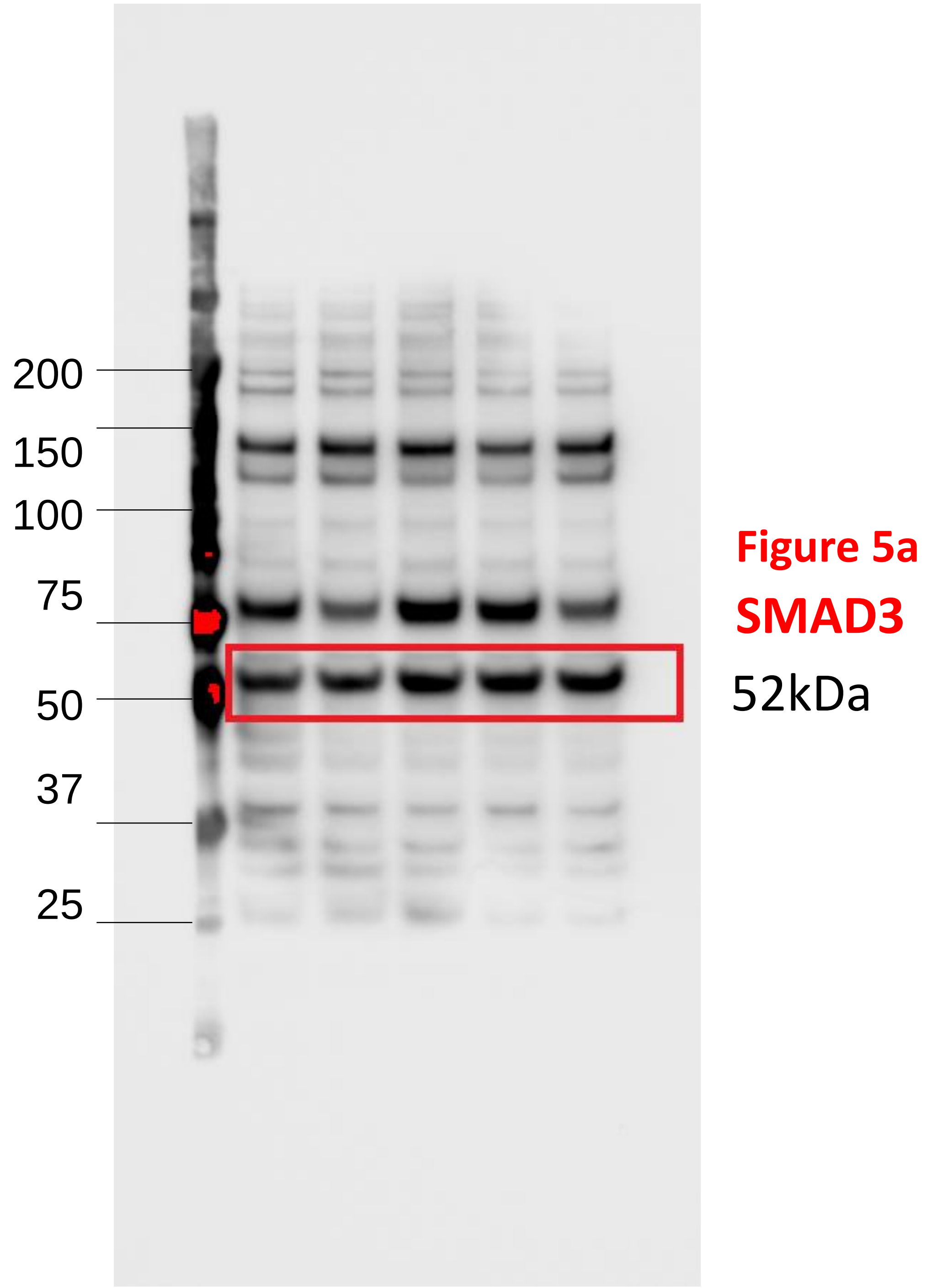

200
150
100
75
50
37
25
Figure 5a
SMAD3
52kDa

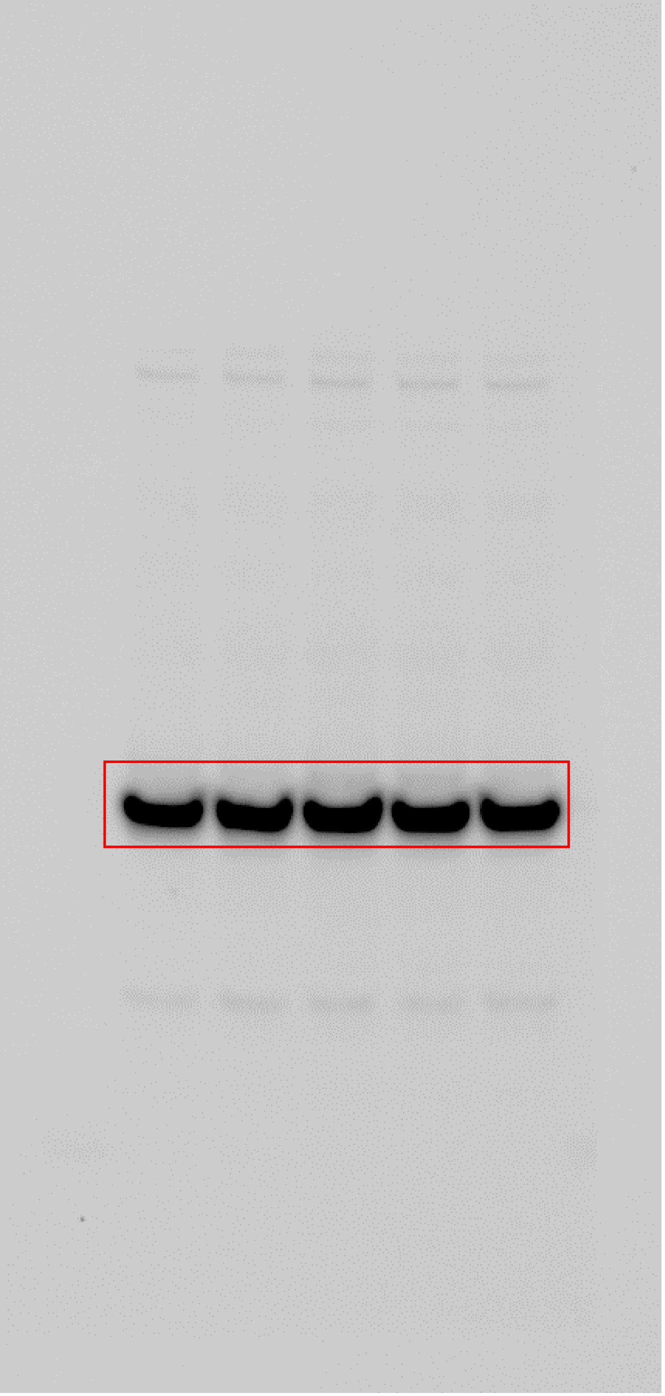

Figure 5a
GAPDH
37kDa

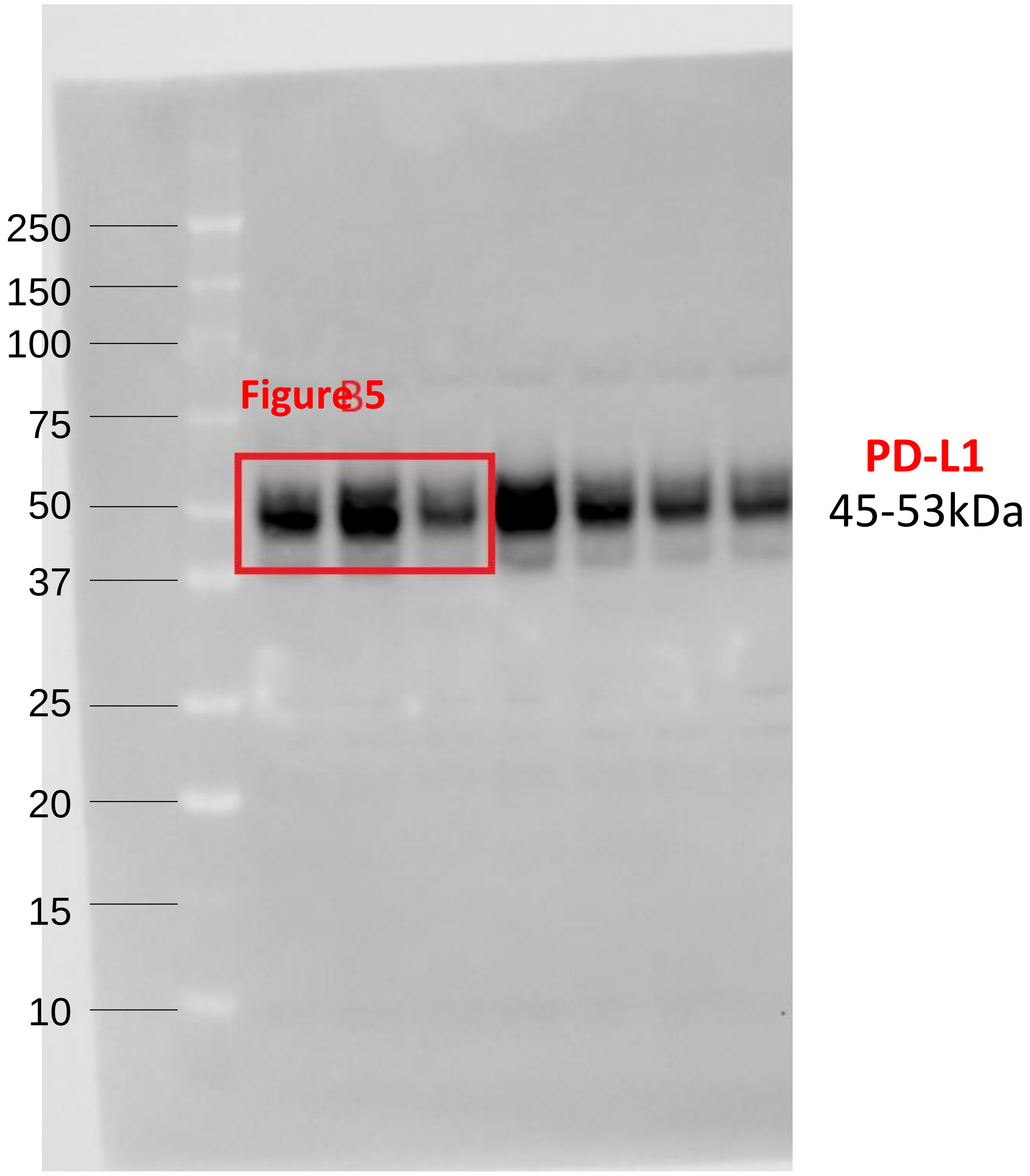
250
150
100
75
50
37
25
20
15
10
Figure 5
PD-L1
45-53kDa

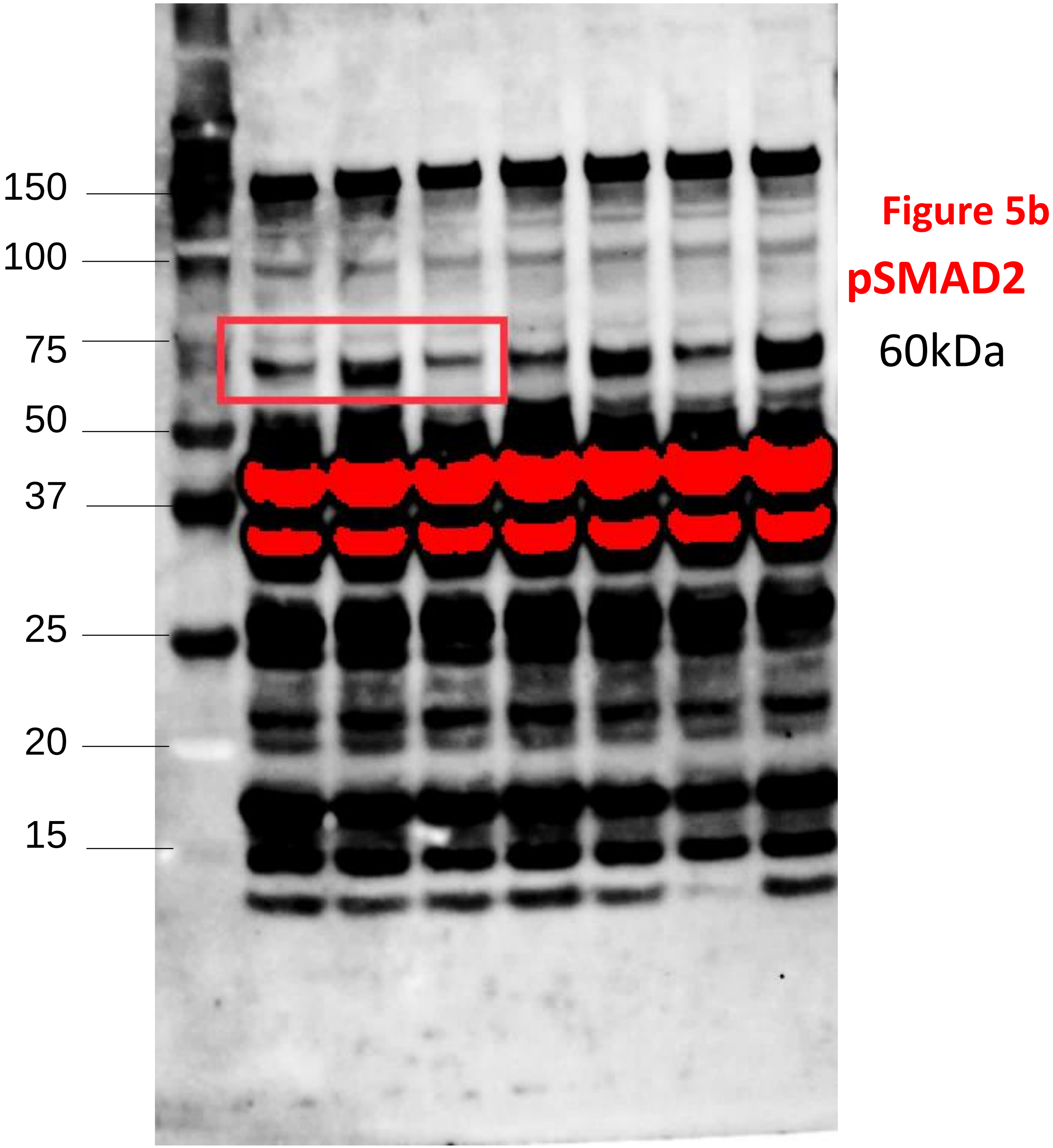
150
100
75
50
37
25
20
15
Figure 5b
pSMAD2
60kDa

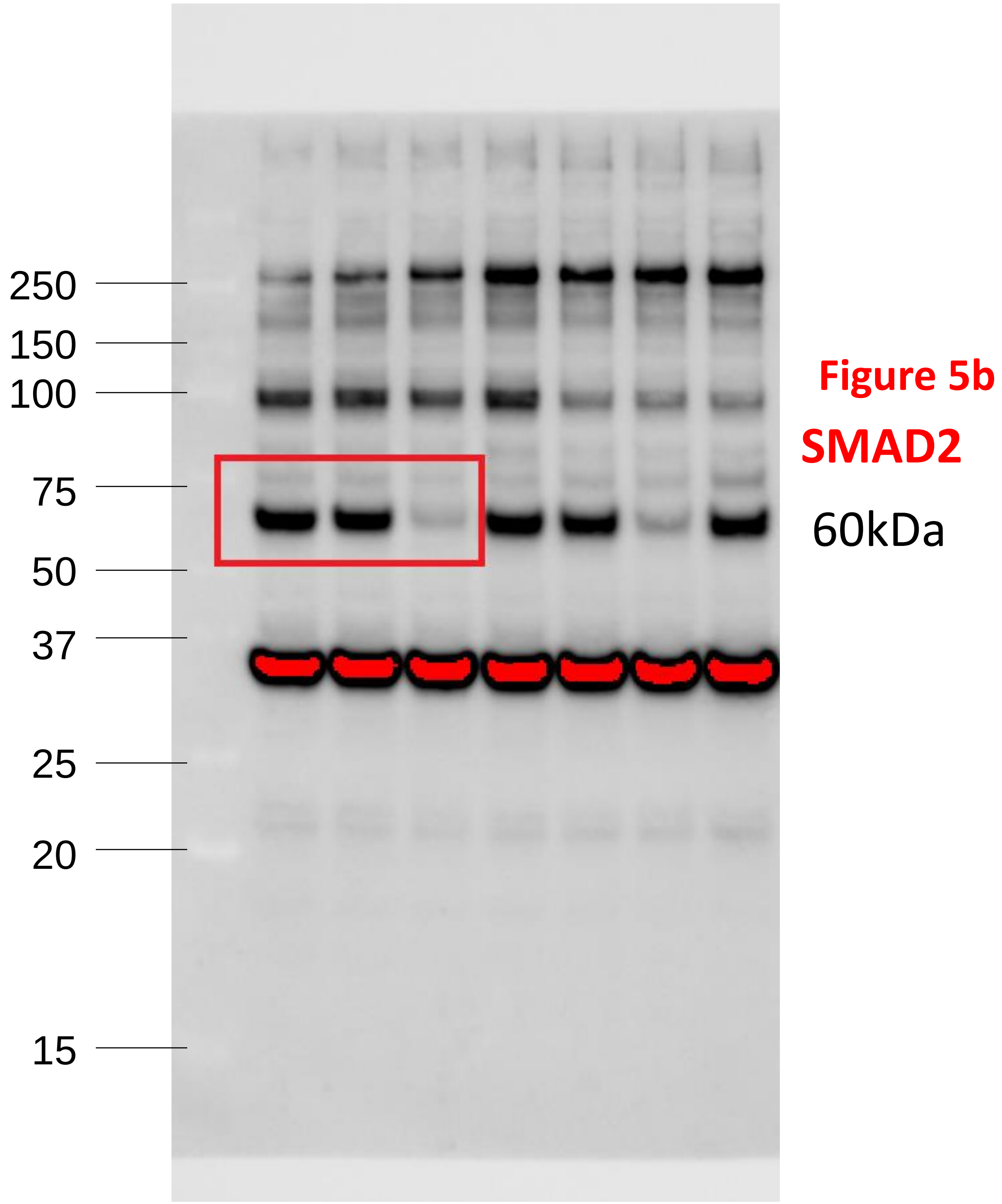
250
150
100
75
50
37
25
20
15
Figure 5b
SMAD2
60kDa

Figure 5b

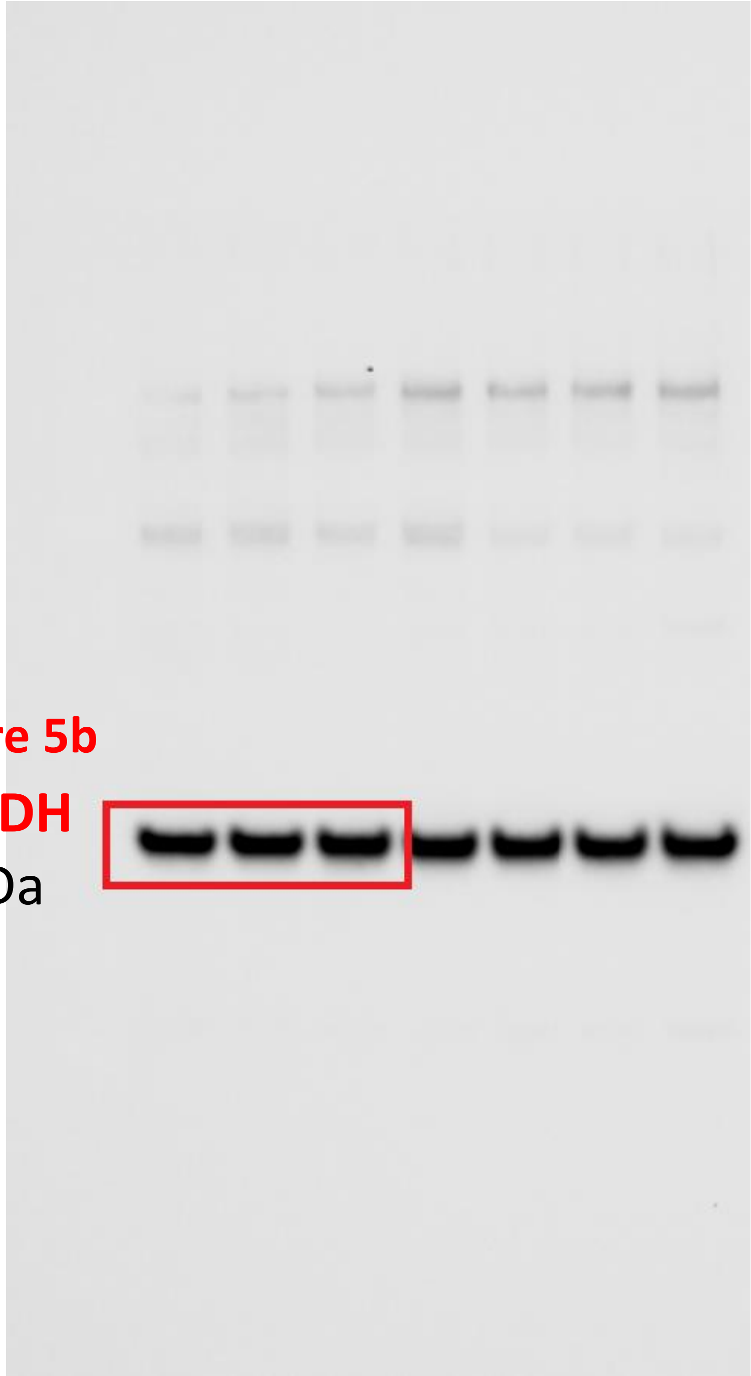

Supplement: Supplementary file 2 — Supplementary Figures 1-16 [file 41698_2024_523_MOESM2_ESM.pdf]
